# Characterization of Natural Organic Matter and Humic Substance Isolates by Size Exclusion Chromatography following Reduction with Sodium Borohydride

**DOI:** 10.1021/acsenvironau.4c00075

**Published:** 2024-12-20

**Authors:** Hang Li, Blair Hanson, Garrett McKay

**Affiliations:** † Zachry Department of Civil & Environmental Engineering, Texas A&M University, College Station, Texas 77843, United States; ^‡^Department of Civil, Environmental, and Architectural Engineering and ^§^Environmental Engineering Program, University of Colorado Boulder, Boulder, Colorado 80303, United States

**Keywords:** dissolved organic matter, optical properties, sodium borohydride, redox, molecular size

## Abstract

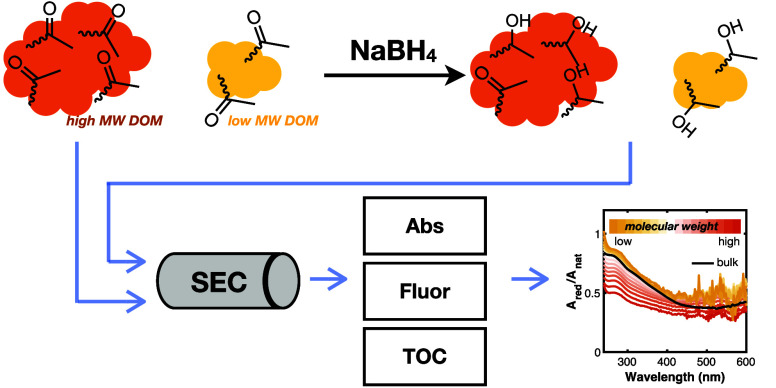

Chemical reduction with sodium borohydride has been used for over four decades to probe the presence and function of carbonyl-containing moieties in dissolved organic matter (DOM). One of these structure–property relationships is the attenuation of UV–visible absorbance after borohydride reduction, an effect that has been observed universally across DOM of different origins. We previously demonstrated that DOM with similar bulk physicochemical properties exhibits bifurcating reactivity with borohydride depending on the source (i.e., soil vs. aquatic), as judged by the kinetics of fractional absorbance removal during reduction at a fixed borohydride:DOM mass ratio. This result and data from other studies suggest that a portion of borohydride-reducible chromophores in DOM may be inaccessible to the water solvent, explaining the incomplete absorbance attenuation even at very high borohydride mass excesses. Here, we study the reactivity of five DOM isolates with sodium borohydride via size exclusion chromatography coupled to total organic carbon, absorbance, and fluorescence detectors. Reduction with sodium borohydride resulted in quantifiable yet exceedingly small decreases in DOM molecular weight, suggesting that the reduction of carbonyl groups to alcohols does not markedly impact the DOM secondary structure. Interestingly, higher molecular weight DOM exhibited the most prominent changes in optical properties after reduction, suggesting that larger molecules contain a high proportion of borohydride-reducible moieties. Optical surrogates were inversely correlated to molecular weight across a single isolate, both native and reduced. However, the correlation broke down at lower molecular weights, wherein optical surrogates remained constant with continued decreases in elution volume, suggesting that there is an intrinsic lower limit to the ability of optical surrogates to capture further decreases in molecular weight. Overall, these results provide insights into the DOM structure and help inform future applications of sodium borohydride for studying the DOM source and reactivity.

## Introduction

Naturally occurring dissolved organic matter (DOM) is a mixture of compounds that are present in all water bodies and plays critical roles in natural and engineered systems.^1−3^ The role of DOM in these systems is, in part, governed by its chemical and physical structure; however, efforts to resolve DOM structure have been challenged by its heterogeneity and molecular complexity.^1,4^ For example, although ultrahigh resolution mass spectrometry reveals that DOM contains many thousands of different molecules,^5−8^ resolving precise chemical structure from these data requires additional experiments or analyses.^9−11^

One way to probe DOM’s chemical composition involves selective transformation of key functional groups.^11−13^ Monitoring the DOM property of interest before, during, and after such reactions provides a direct link between the chemistry of that transformation and observable properties, provided that appropriate controls are performed to test for the potential impact of reaction conditions. A nonexhaustive list of chemical transformations used to study structure–function relationships in DOM includes ozone,^14−16^ reduction with metals^17,18^ and electrodes,^19−21^ acetylation,^22^ and proton exchange.^11,23,24^

**Scheme 1:**
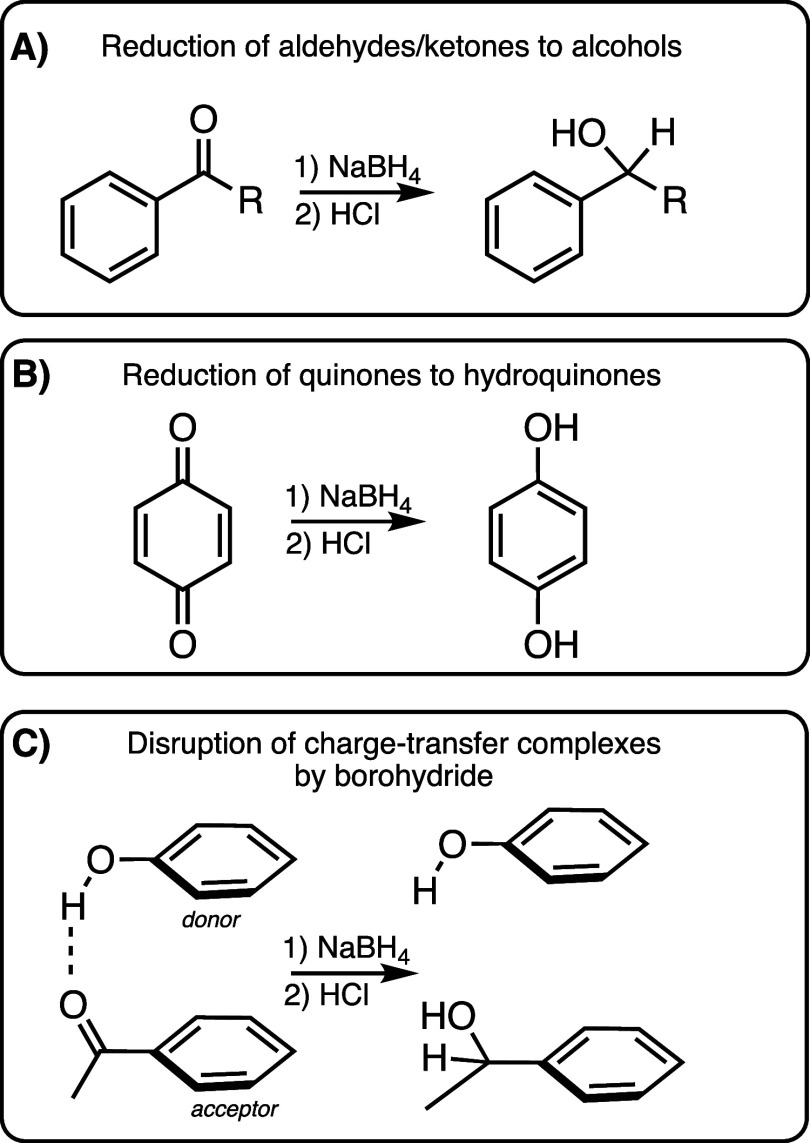
Influence of Borohydride Reduction on Electron Acceptor Moieties, Including Ketones/aldehydes (A) and Quinones (B). Borohydride Reduction of Carbonyl-containing Acceptors is Hypothesized to Disrupt Charge-Transfer Interactions in DOM (C)

One of the most common transformations, spanning over four decades of use, is chemical reduction with sodium borohydride (NaBH_4_). Borohydride is a common reagent used in organic chemistry for reducing ketones and aldehydes to their corresponding alcohols (Scheme 1). The initial step in the reaction involves hydride transfer from the nucleophilic borohydride (BH_4_^–^) to the electrophilic C=O group, with the alcohol formed upon acidic workup. In an early study, Leenheer et al. used NaBH_4_ to help infer the predominance of aromatic ketone’s contribution to C=O groups in humic substance isolates from the Suwannee River.^25^ Tinnacher and Honeyman later used tritiated borohydride as a radiolabeling technique, demonstrating via size exclusion chromatography (SEC) that ^3^H was uniformly distributed over the molecular size range of Suwannee River fulvic acid.^26^ Pioneering work by Del Vecchio, Blough, and colleagues studied the impact of borohydride reduction on absorbance and fluorescence spectra of DOM and humic substances.^27^ These studies and others have demonstrated a consistent response of optical properties to borohydride reduction–preferential attenuation of visible absorption, increased and blue-shifted fluorescence emission, and increasing fluorescence quantum yields–for a diverse set of humic substance isolates,^27,28^ marine whole waters and isolates,^29−31^ and other complex carbonaceous materials (e.g., atmospheric brown carbon).^32^ More recently, studies combining mass labeling (using NaBD_4_) have demonstrated that >30% of formulas detected by negative mode electrospray ionization Fourier transform ion cyclotron resonance mass spectrometry contain a borohydride-reducible moiety.^33^ Although DOM can contain both aliphatic and aromatic carbonyls,^34^ the latter are likely more important chromophores in sunlit waters.^35,36^ Taken together, these prior studies indicate carbonyl moieties play an important role in DOM chemistry.

Results from NaBH_4_ reduction have been broadly used to support a photophysical model in which interacting chromophores play a key role in the emergence of DOM optical and photochemical properties. In this model, electron-poor acceptor moieties (e.g., aromatic aldehydes and ketones, or quinones) interact in their ground or excited electronic state with electron-rich donor moieties (e.g., phenols, alkoxy phenols, and polyphenols).^37−39^ Transformation of carbonyl-containing electron acceptors to their corresponding alcohols removes these moieties’ ability to serve as electron acceptors. Loss of such donor–acceptor complexes is consistent with the changes observed after NaBH_4_ reduction, including increases in spectral slope (less visible absorbance due to charge-transfer) and blue-shifted and enhanced fluorescence emission (increased emission from local excited states due to a decreased rate of excited state proton/electron transfer). It has been argued that the impacts of NaBH_4_ reduction are inconsistent with reduction of isolated chromophores and fluorophores and that only an electronic interaction model can explain these aggregate observations.^27,38,40^

In prior work seeking to elucidate the role of charge-transfer interactions on the optical properties of DOM, McKay, Korak et al. demonstrated that absorbance and fluorescence spectra of DOM were largely independent of solvent polarity and temperature, which was taken as evidence that donor–acceptor complexes do not play a significant role in DOM optical properties.^41^ In response, Del Vecchio and Blough hypothesized that charge-transfer complexes in DOM were thermodynamically stable and isolated from solvent (and therefore not in contact with organic solvent) because they were encapsulated in a hydrophobic DOM core.^40,42^ They further argued that the incomplete absorbance attenuation of humic substances during NaBH_4_ reduction even at very high borohydride doses is evidence for encapsulated charge-transfer complexes.^12,40^ A corollary to this hypothesis is that donor–acceptor complexes themselves may play a role in modulating DOM structure. Indeed, past research using SEC,^43,44^ NMR,^45^ and molecular dynamics simulations^46,47^ has suggested that noncovalent interactions between smaller molecules may contribute to DOM three-dimensional structure. Based on this prior literature, we hypothesized that chemical reduction of DOM with NaBH_4_, which could disrupt charge-transfer contacts, would result in DOM with a lower molecular weight. Isolated charge-transfer complexes may also lead to unique dependence of optical properties of DOM on molecular weight due to varying accessibility of donor–acceptor pairs or differences between fractions in the abundance of borohydride-reducible groups.

To test this hypothesis, we reduced five DOM isolates with NaBH_4_ and employed SEC to characterize the size distribution of total organic carbon (TOC), absorbance, and fluorescence in native and reduced samples. In addition, apparent fluorescence quantum yields (AQY) of the native and reduced fractions were calculated as a function of elution volume using a recently developed methodology.^48^ Spectral changes observed during SEC were compared to those for bulk samples. In addition to testing the impact of borohydride reduction on the molecular weight of DOM, these measurements allow us to examine the size dependence of the borohydride-induced changes in optical properties. Overall, results and analyses from this study provide insight into the distribution of borohydride-reducible groups in DOM, informing future applications of NaBH_4_ for differentiating sources and reactivity of DOM and other complex mixtures.

## Materials and Methods

### Chemicals and DOM Samples

Humic substance and natural organic matter (NOM) isolates were obtained from the International Humic Substances Society, including Elliot Soil humic acid (ESHA 5S102H), Elliot Soil fulvic acid (ESFA 5S102F), Suwannee River humic acid (SRHA 3S101H), Suwannee River fulvic acid (SRFA 3S101F), and Suwannee River natural organic matter (SRNOM, 2R101N). Solutions for reduction experiments and the control group were prepared at 200 mg/L in pH 7 ultrapure water (≥18.2 MΩ-cm, Barnstead Nanopure). NaBH_4_ stock solution (5 g/L) were made in water preadjusted to pH 12 with NaOH. All humic substance and NOM solutions were filtered with 0.45 μm poly(ether sulfone) syringe filters (Supor) right before the reaction with borohydride. The filters were prerinsed with 20 mL of ultrapure water.

### Borohydride Reduction

For the reduction group, 25-fold mass excess of NaBH_4_ was added to the DOM samples and fully stirred.^12^ The control group was prepared at the same DOM concentration but without addition of NaBH_4_. Both the reduction and control group solutions were adjusted to pH 10 with 2 M NaOH and maintained at this pH for 4 days, in the dark. Although past work has shown minimal changes in spectral shape and intensity of bulk samples during high pH exposure,^28^ the control experiment done here ensures that potential changes in spectra across the size gradients produced in SEC are due to reaction with NaBH_4_ and not hydroxide ions. After 4 days, both groups were adjusted to pH 7 with 2 M HCl to quench residual borohydride.

### Analytical Methods

The reduction group and control group samples were diluted to 20 or 40 mg L^–1^ in 0.01 M phosphate buffer of pH 6.8 and filtered with water-rinsed 0.45 μm poly(ether sulfone) syringe filters. Absorbance and fluorescence spectra were measured on diluted (bulk) samples using an Aqualog (Horiba). The excitation wavelength (λ_ex_) ranged from 240 to 800 nm (in 5 nm increments) and the emission wavelength (λ_em_) ranged from 250 to 800 nm (bin setting of 2.33 nm). The sample integration time was 0.2 or 0.5 s depending on the sample absorbance and emission intensity.

Aliquots of the diluted samples were shipped overnight to CU Boulder (Boulder, CO) in ice-packed coolers for SEC analysis as described previously.^48^ Briefly, the in-line SEC system employed a Toyopearl HW-50S column with a mobile phase of phosphate buffer with sodium sulfate of pH 6.8, 0.1 M ionic strength, at a flow rate of 1 mL min^–1^. Samples (1.8 mL, 10 mg L^–1^) were adjusted to the eluent ionic strength (0.1 M) prior to injection using a concentrated mobile phase solution. Following elution from the SEC column, samples passed through multiple detectors, including an Agilent 1260 Infinity Series G1315D Diode Array Detector (DAD) from 200 to 700 nm with 2 nm increments, 1260 Infinity II Series G7121B Fluorescence Detectors (FLD) at λ_ex_ = 350 nm and λ_em_ = 350–700 nm with 5 nm increments, and a Sievers M9 TOC Analyzer. The TOC analyzer utilizes UV/persulfate oxidation and was calibrated with standard solutions of potassium hydrogen phthalate (KHP). The SEC run time lasted up to 200 min to ensure complete sample elution prior to the next injection.

### Data Processing

SEC data included absorbance spectra, fluorescence emission spectra at λ_ex_ = 350 nm, and TOC measured at each elution volume. Several data treatment procedures were performed on the raw data. Signals measured prior to and after elution of DOM were averaged and used for blank subtraction and to correct for baseline drift in the case of SEC-absorbance data. Fluorescence data were further treated for instrument-specific correction factors, inner filter corrections, and masking of first order Rayleigh scatter. Although emission spectra were collected on the SEC out to 700 nm, they were trimmed at >600 nm due to low signal-to-noise ratios for some samples and large second order Rayleigh scatter. AQY as a function of elution volume were calculated according to Hanson et al.^48^ Briefly, the method utilizes salicylic acid (quantum yield of 0.36 at 300 nm excitation),^49^ dissolved in the same mobile phase used for sample analysis, as a fluorescence reference (note that salicylic acid was also used for detector alignment). One limitation of AQY calculations is that, because emission data is trimmed, a portion of the fluorescence signal (emission >600 nm) cannot be integrated. This results in a lower integrated fluorescence intensity and consequently a lower AQY. SRHA and ESHA, which have the most red-shifted fluorescence spectra, would have the greatest impact from this limitation.

Because TOC, absorbance, and fluorescence detectors acquired data at different frequencies, chromatographic data were interpolated to achieve a uniform elution volume increment (0.5 mL). A cubic spline interpolation was tested against other methods and found to give the lowest residuals, with smoothing parameters of 1, 0.95, and 0.90 used for spectra, absorbance chromatograms, and fluorescence chromatograms, respectively.

Bulk sample fluorescence spectra were corrected using standard procedures,^50^ including blank subtraction, inner filter corrections, Rayleigh and Raman masking, and Raman normalization.

### Quality Control Criteria

Quality control criteria were developed for both chromatographic and spectral data. Spectral data between 0 and 20 mL elution volume were used as the baseline. The quality check threshold is determined to be 20 times the 95% cumulative background noise, which is the standard deviation of baseline data. For fluorescence data, inner filter and instrument-specific correction factors were used to calculate non corrected data prior to determining the background noise threshold. Several other methods were attempted for fluorescence quality control, which are described in SI Text S1. Chromatograms and spectra with a maximum signal lower than the threshold were excluded from subsequent data analysis. To further filter noisy data, summed intensities at each elution volume that were less than 10% of the maximum summed intensity were excluded.^51^

### Data Analysis

In addition to qualitative analysis of chromatograms, several metrics were calculated to evaluate quantitative differences between the control and reduction group. Inspired by Wünsch et al.,^52^ we calculated Tucker Congruence Coefficients (TCC)^53,54^ and Shape Sensitive Congruence (SSC) to compare chromatograms of the native and reduced samples. TCC describes the difference in peak position between two chromatograms (eq 1)
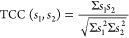
1where *s*_1_ and *s*_2_ represent chromatograms to be compared (e.g., native and reduced) with identical *x*-axes. SSC is thought to be a more sensitive quantification of peak differences by including penalty terms α and β that represent the difference in maximum position and area of the two peaks (eq 2)
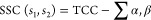
2where
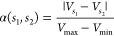
3
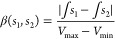
4In eqs 3 and 4, |*V*_*s*_1__–*V*_*s*_2__| is the difference in peak position between *s*_1_ and *s*_2_ in milliliters, *V*_max_–*V*_min_ represents the range of observed elution volume in milliliters, and |∫*s*__1__–∫*s*__2__| is the difference in areas of *s*_1_ and *s*_2_ normalized to their respective maximum peak intensities.

Although TCC > 0.95 is considered a “match” for PARAFAC components, there is no such accepted threshold for SSC, which tends to be lower than TCC,^52^ nor is there an accepted threshold for comparing SEC chromatograms. As a first approximation, we derived threshold TCC and SSC values from replicate chromatograms of SRFA, SRHA, and SRNOM (*n* = 4 per sample, *n* = 12 total). Complementing this replicate analysis, we also compared chromatograms of native DOM samples that been passed through a column of Sephadex G-10 resin to remove borate salts, which is not expected to modify DOM molecular weight (see Text S2), but has been employed previously in borohydride reduction workflows.^12^

Additional quantitative metrics for comparing spectra included the intensity-weighted shift in wavelength, which was calculated for both absorbance and fluorescence spectra as a function of elution volume (eqs 5 and 6). *A*_red,*i*_ and *A*_nat,*i*_ are the absorbance at each absorbance wavelength (λ_*i*_) of the reduced and native samples, respectively. *F*_red,*i*_ and *F*_nat,*i*_ are the corresponding fluorescence data at λ_ex_ = 350 nm, and each λ_em_. These parameters describe the increase in steepness of absorbance spectra and decrease in peak emission maxima for the reduced sample compared to the control.
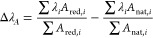
5
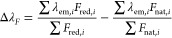
6

Finally, optical surrogates were calculated as a function of elution volume. The first, *A*_250_/*A*_364_, is nearly identical to the widely used E2:E3 ratio (defined as *A*_250_/*A*_365_).^55,56^*A*_250_/*A*_364_ was used instead of E2:E3 because the SEC absorbance detector wavelength increment was 2 nm. The second optical surrogate is closely related to the fluorescence index (FI),^57,58^ which is defined as the ratio of fluorescence emission at 470 to 520 at 370 nm excitation. In this study, the SEC fluorescence detector was set at 350 nm excitation and the ratio of fluorescence emission at 470 to 520 nm was calculated (*F*_470_/*F*_520_). While 370 nm has been used in previous studies of bulk DOM fluorescence, SEC results in a significant dilution of samples. The choice of 350 nm relates to the absorbance signal needed for AQY calculations where a lower absorbance threshold of 0.5 mAu was used as a QA/QC measure, below which AQY is not calculated. Because the absorbance spectra of DOM are generally characterized by an exponential decrease with increasing wavelength, 350 nm provides a greater absorbance signal and extends the range of elution volumes for which AQY is calculated.

## Results

### Typical Chromatograms for TOC, Absorbance, and Fluorescence Detectors

This study presents chromatograms with elution volume on the *x*-axis. Elution volume is proportional to retention time (elution volume = retention time × flow rate) and, in SEC, is inversely proportional to molecular size. Although SEC studies often convert column retention time to molecular weight using polymer standards, we chose not to do so (detailed explanation presented in Text S3). The term “molecular weight” will be used in the later sections to describe the distribution of DOM fractions presented by retention time, while recognizing that SEC separates by size.

**Figure 1:**
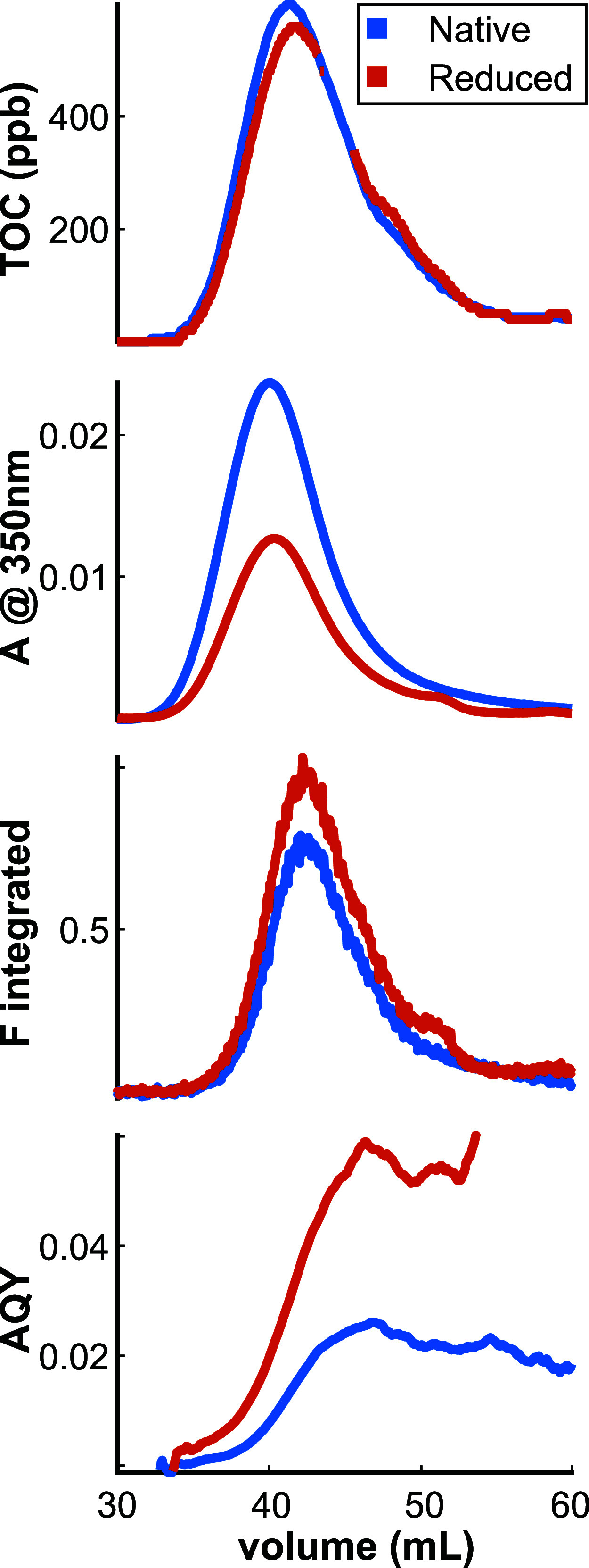
Chromatography of SRFA. From top to bottom: TOC, absorbance at 350 nm, integrated fluorescence emission at 350 nm excitation, and fluorescence quantum yield vs elution volume.

Figure 1 shows SEC chromatograms for SRFA measured by TOC, absorbance, and fluorescence detectors. Although we use the term TOC, the sample was filtered through 0.45 μM filter prior to injection and thus the signal is attributable to dissolved organic carbon. The absorbance chromatogram shown in Figure 1B is at a wavelength of 350 nm. The fluorescence detector was set to 350 nm excitation and Figure 1C represents the integrated fluorescence emission. Finally, AQY values shown in Figure 1D are calculated utilizing both absorbance and fluorescence data.

Peak elution volumes for TOC occur just before absorbance and the fluorescence peak elution volume occurs after absorbance, consistent with a prior study of SRFA.^48^ Because of the elution volume offset between absorbance and fluorescence, AQY values are highest at elution volumes larger than the peak of both absorbance and fluorescence chromatograms.

Figure 1 also demonstrates the typical impacts of NaBH_4_ reduction observed in SEC data that will be evaluated in detail below. Native and reduced SRFA exhibit highly similar TOC chromatograms, with reduced SRFA slightly shifted to larger elution volumes (lower molecular weight). The absorbance chromatogram of reduced SRFA is ∼50% lower in intensity at the peak than native SRFA, with integrated fluorescence being larger for the reduced sample. Taken together, these changes result in an increase in AQY at all elution volumes for reduced compared to native SRFA. A notable observation from these data is that optical changes occur across a range of elution volumes (large to small), indicating that SRFA has borohydride-reducible moieties that contribute to optical signals across a range of molecular weights.

### Impact of NaBH_4_ Reduction on TOC Chromatograms

One objective of this study was to test the hypothesis that intermolecular electron donor–acceptor complexes contribute to the three-dimensional structure of DOM (Scheme 1C), as proposed by Blough and Del Vecchio.^40^ Because SEC with TOC detection provides an evaluation of the size distribution of all carbon-containing molecules in DOM, we reasoned that comparing TOC chromatograms of native and reduced samples would be a good test of this hypothesis. If NaBH_4_ reduction disrupts donor–acceptor complexes, TOC chromatograms would be expected to shift toward larger elution volumes (lower molecular weight). This hypothesis was tested using both a qualitative comparison of the chromatograms and a more quantitative approach utilizing TCC and SSC.^52^

**Figure 2:**
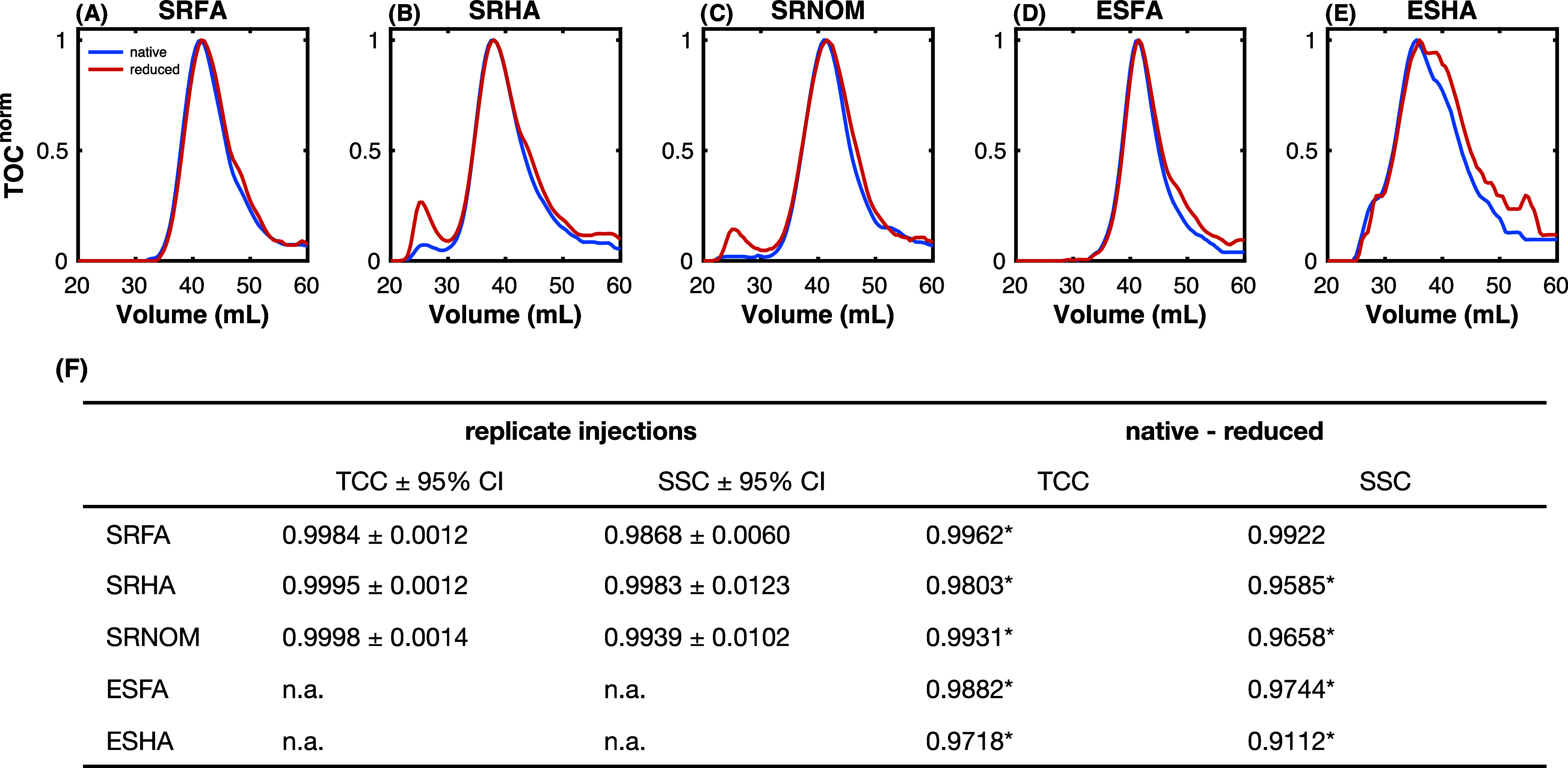
TOC chromatograms of native and reduced DOM isolates normalized to peak intensity, including (A) Suwannee River fulvic acid (SRFA), (B) Suwannee River humic acid (SRHA), (C) Suwannee River natural organic matter (SRNOM), (D) Elliot Soil fulvic acid (ESFA), and (E) Elliot Soil humic acid (ESHA). (F) Tucker Congruence Coefficient (TCC) and size sensitive congruence (SSC) for samples under different conditions. SSC is equal to TCC subtracted by penalization terms that consider differences in peak area and peak position. The replicate injections label evaluates the difference between replicate native samples. TCC ± 95% CI and SSC ± 95% CI indicate 95% confidence intervals from four replicates. The native–reduced label evaluates the impact of NaBH_4_ reduction. An asterisk indicates that the value falls below the lower limit of the corresponding 95% CI.

Figure 2A–E show that the primary peak of reduced sample’s TOC chromatograms is slightly shifted to larger elution volumes. Although small, the shift to larger elution volume for reduced samples is statistically significant, indicating the possibility that borohydride reduction decreases DOM molecular weight. Importantly, native samples were controlled by maintaining solutions at pH 10 for 4 days (the equivalent pH of borohydride reduced samples) to account for the potential of hydroxide-catalyzed reactions to decrease molecular weight.

Unique to SRHA and SRNOM is the appearance of a peak at ∼27 mL at lower elution volumes that is either absent or less apparent in the native chromatogram. It is important to note that potential complexation of residual borate ions, which were not removed prior to SEC analysis (see Text S2), has been shown to decrease DOM’s electrophoretic mobility.^59^ Such complexations would likely cause an increase in molecular size, which may explain the ∼27 mL peak for SRHA and SRNOM.

TCC and SSC values for the native-reduced group (treatment) TOC chromatograms are shown in Figure 2F and are compared to the lower 95% confidence interval of the equivalent values for native replicates (control). The replicates are derived from multiple injections of the same samples, which is considered a threshold for evaluating TCC and SSC of DOM samples. All TCC values for the treatment group are lower than the control TCC (<0.997). Considering SSC, four out of five values for the treatment group are lower than the control, with SRFA (SSC of 0.992) being the exception.

### Comparison of Native and Reduced Spectra for Bulk DOM and Size-Separated DOM at the Peak Elution Volume

**Figure 3:**
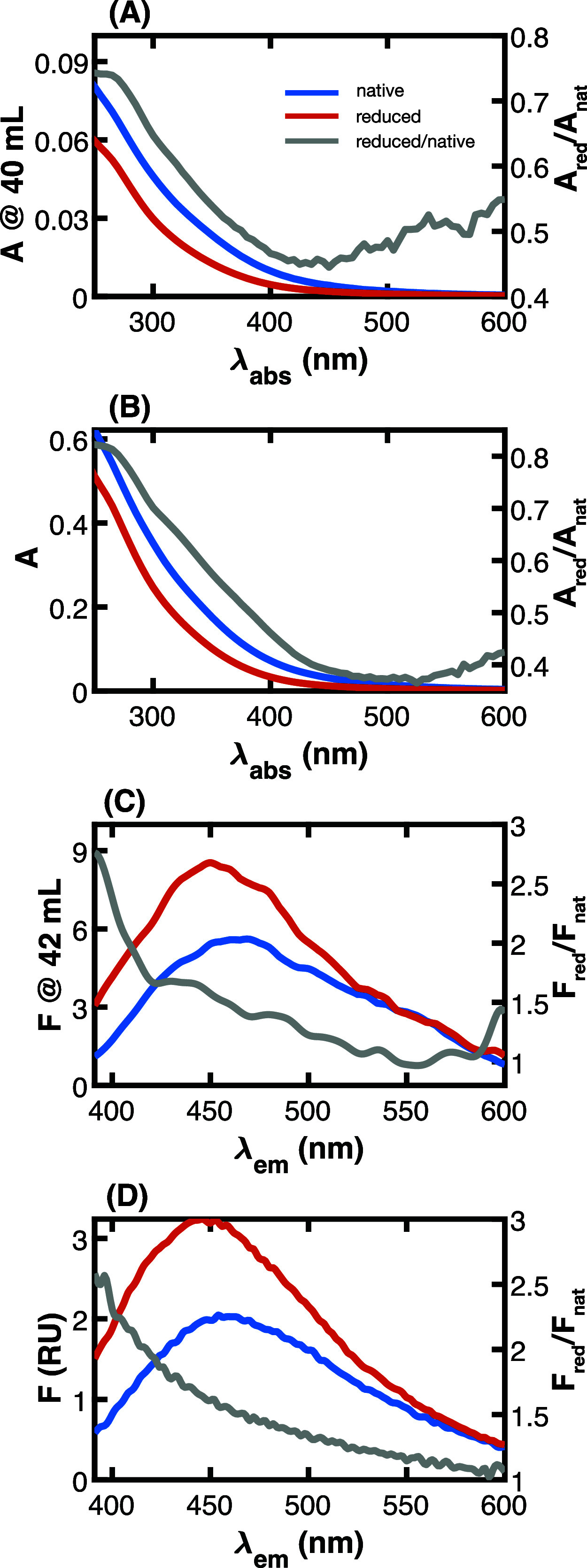
Absorbance (A and B) and fluorescence spectra (C and D) of Suwannee River fulvic acid at the peak elution volume (A and C) and bulk samples (B and D). The right *y*-axis (gray lines) shows the ratio of reduced to native spectra. Spectra in (D) are normalized to water Raman scattering, whereas units in (C) are arbitrary. Emission spectra in (C) and (D) are collected at 350 nm excitation.

Before presenting the dependence of optical spectra on elution volume, it is instructive to show spectra for native and reduced SRFA collected for the bulk sample and at the peak elution volume (∼40 mL) during SEC (Figure 3). The spectral shape before and after borohydride reduction are qualitatively similar for bulk and SEC measurements. Absorbance spectra decrease in intensity across the UV–visible range following borohydride reduction, concomitant with an increase and blue-shifted fluorescence emission. Notably, SRFA’s red-edge emission (>550 nm) is largely unaltered after reduction with NaBH_4_. There is also good quantitative agreement in the magnitude of bulk samples’ optical changes. The fractional absorbance removal, *A*_red_/*A*_nat_, reaches a minimum of ∼0.5 for spectra collected at the peak emission volume; a similar minimum (∼0.4) is observed for the bulk sample. The lower *A*_red_/*A*_nat_ observed for bulk SRFA may also be due to the different signal-to-noise ratios of different instruments. The ratio of reduced to native fluorescence emission, *F*_red_/*F*_nat_, shows similar agreement, with maximum values near 2.5 to 3 at ∼400 nm and a minimum of ∼1 at ∼550 nm.

Like results for SRFA, good qualitative and quantitative agreement is observed for spectra and their fractional change after NaBH_4_ reduction for other DOM isolates (Figure S1). Notable exceptions are that *A*_red_/*A*_nat_ for SRNOM and SRHA tend to be lower for bulk samples compared to *A*_red_/*A*_nat_ at the peak elution volume.

### Impact of NaBH_4_ Reduction on DOM Absorbance Spectra and Chromatograms

**Figure 4:**
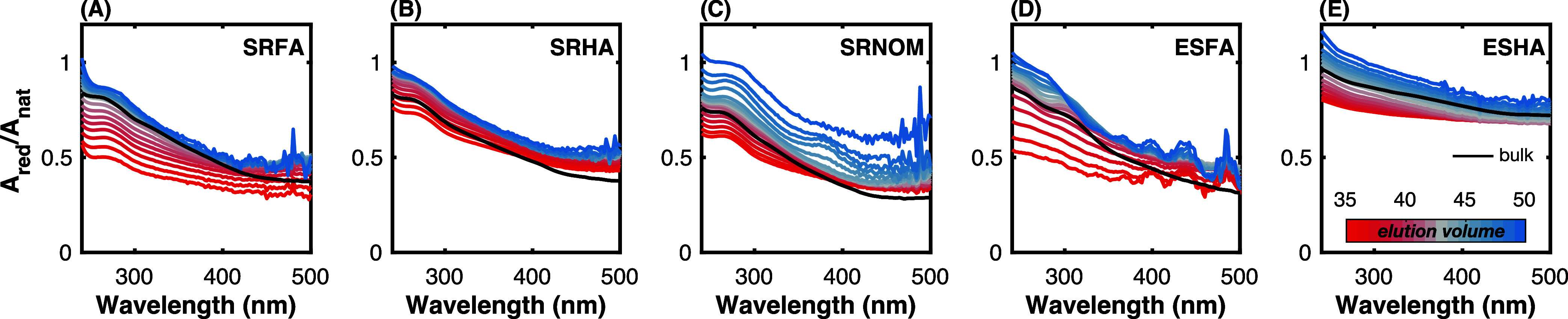
Dependence of fractional absorbance (*A*_red_/*A*_nat_) remaining on elution volume. Spectra collected at pH 7. NaBH_4_ reduction carried out for 4 days at 25-mass fold excess NaBH_4_. Heatmap: 35 to 50 mL elution volume. *A*_red_/*A*_nat_ of the bulk sample is indicated by a solid black line.

NaBH_4_ reduction of all DOM isolates results in loss of UV and visible absorbance across a range of elution volumes (35 to 50 mL, Figure 4, Figure S2). *A*_red_/*A*_nat_ was lowest for elution volumes below ∼42 mL. In contrast, *A*_red_/*A*_nat_ for SRFA, SRHA, and ESFA remained largely the same at elution volumes above ∼45 mL (Figure 4A,B, and D). These results imply that material at a range of molecular weights (small to large) contains borohydride-reducible moieties, but that these groups play a more significant role in the absorbance of high molecular weight DOM. The inverse relationship between molecular weight and *A*_red_/*A*_nat_ (higher molecular weight, lower *A*_red_/*A*_nat_) agrees with a prior study showing that *A*_red_/*A*_nat_ for size-fractionated (with a 5 kDa ultrafiltration membrane) SRFA decreased in the order SRFA < 5K, SRFA bulk, SRFA > 5K.^60^

**Figure 5:**
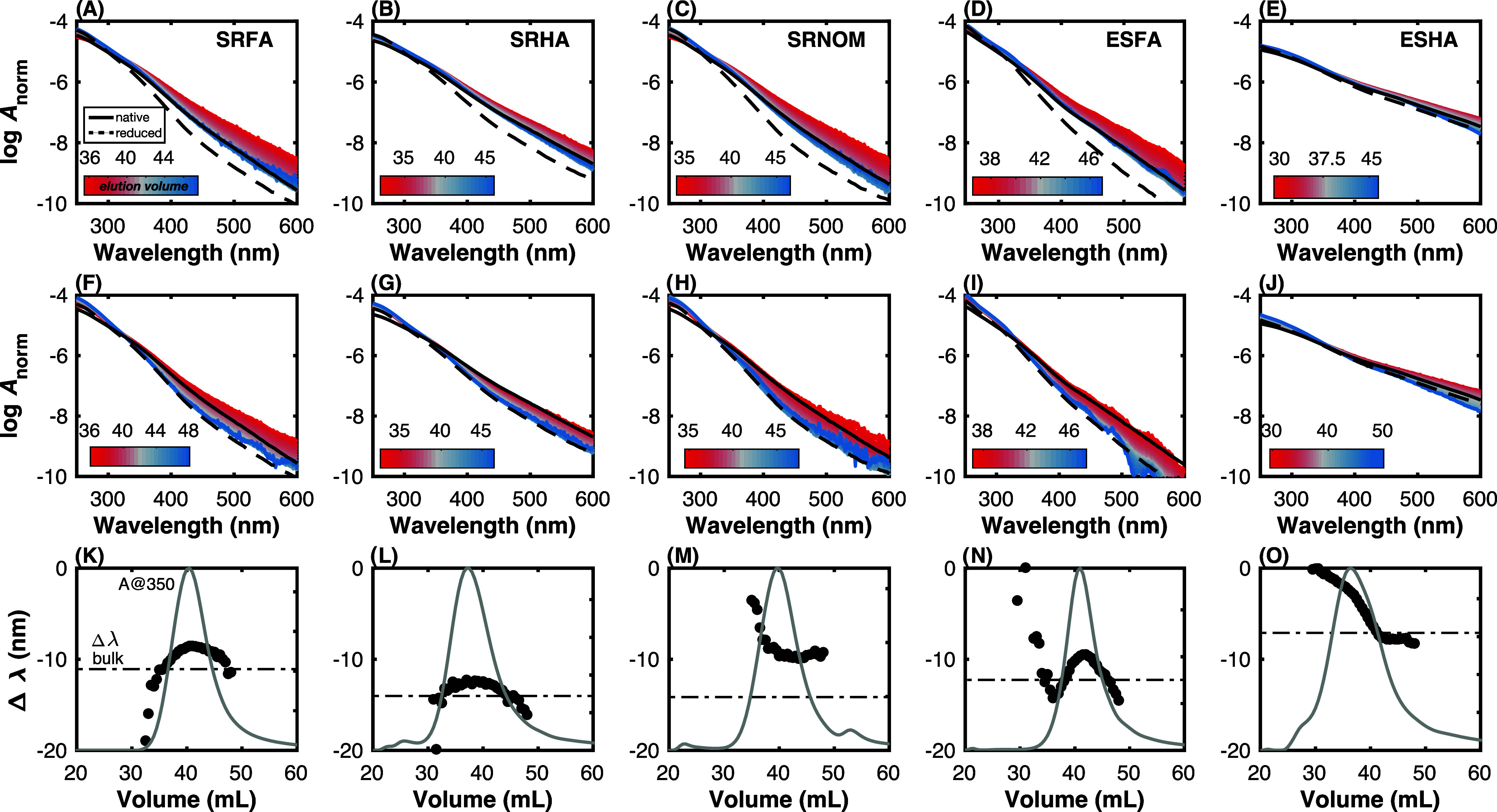
Absorbance properties of DOM isolates as a function of molecular weight and reduction with NaBH_4_. Logarithm of area-normalized absorbance spectra (log *A*^norm^) of (A–E) native and (F–J) reduced samples, with the heatmap indicating elution volume. The solid and dashed lines in (A–J) represent the absorbance spectra of the native and reduced bulk samples, respectively. (K–O) Intensity-weighted difference in absorbance wavelength (Δλ*_A_*) as a function of elution volume. The dashed lines represent Δλ*_A_* for the bulk sample. The chromatogram of absorbance at 350 nm excitation is depicted by the gray line (arbitrary *y*-axis). The sample labels in (A–E) apply to (F–O).

A second observation from Figure 4 is that for selected DOM isolates *A*_red_/*A*_nat_ is less dependent on wavelength for lower elution volumes (higher molecular weight). *A*_red_/*A*_nat_ for ESFA and ESHA at an elution volume of ∼35 mL (red lines in Figure 4D,E) decreases less with increasing wavelength in comparison to material eluting at larger elution volumes (lower molecular weight). This result is further reflected in the dependence of intensity-averaged absorbance wavelength shifts Δλ*_A_* (between native and reduced samples) on elution volume (*vide infra*). Briefly, Δλ*_A_* values for ESHA and ESFA approach zero at lower elution volumes (Figure 5N,O), indicating that absorbance attenuation in high molecular weight material is removed proportionally at different wavelengths.

To further explore the impact of molecular weight and reduction on absorbance spectral shape, Figure 5 shows logarithmic absorbance spectra (normalized to area) collected as a function of elution volume for native (Figure 5A–E) and reduced (Figure 5F–J) samples. Spectra for the bulk native and reduced samples are shown for comparison using solid and dashed black lines, respectively.

The variation in spectral slope as a function of elution volume is largest for SRFA, SRHA, and ESFA and smallest for SRNOM and ESHA. This is the case for both native and reduced samples. It has been demonstrated for many types of DOM that NaBH_4_, in general, results in increased spectral slope.^27−32^ The change in spectral slope after NaBH_4_ reduction at each elution volume, judged here by Δλ*_A_*, is generally in good agreement with the bulk sample. SRNOM is an exception in that the bulk and SEC sample’s Δλ*_A_* differs by ∼5 nm. Most of the variation in spectral slope (for both native and reduced samples) is driven by high molecular weight material (lower elution volume). In contrast, spectra of DOM eluting at larger elution volumes are tightly spaced (blue colors in Figure 5A–J). This observation is consistent with prior studies of highly resolved size fractions of DOM. For example, using SEC coupled to a diode array detector, Wünsch et al.^51^ demonstrated that spectral slopes (*S*_300–600_) of four Swedish lakes had the most variation between ∼1 to 4 kDa whereas *S*_300–600_ values were largely independent of molecular weight below 1000 Da. Similarly, using asymmetric field flow fractionation, Guéguen and Cuss^61^ showed that *S*_275–295_ and *S*_350–400_ were less sensitive to decreasing molecular weight at molecular weights less than 1000 Da relative to spectral slopes measured at >1000 Da.

**Figure 6:**
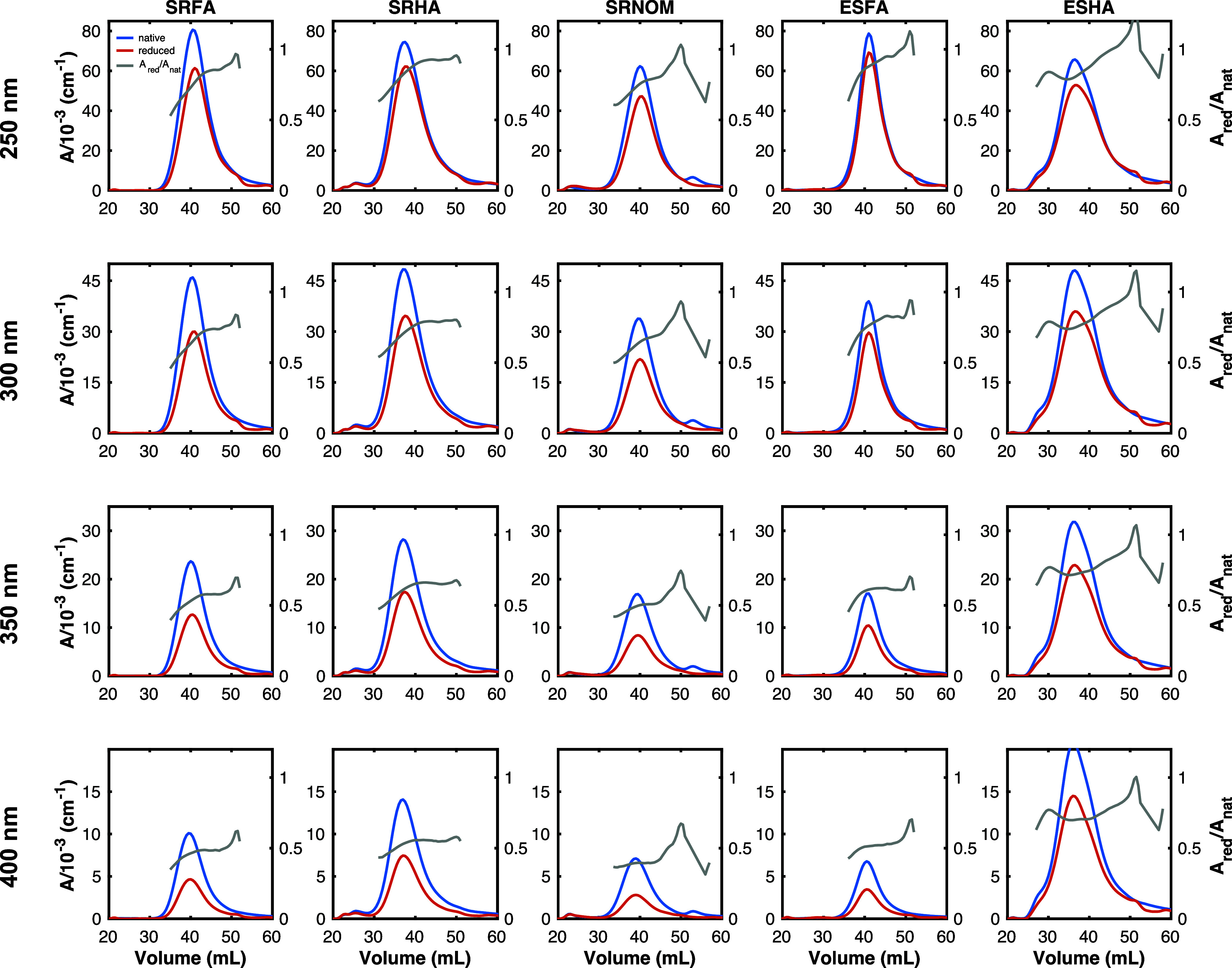
Absorbance chromatograms at multiple wavelengths for all DOM isolates. The gray lines represent the fractional absorbance remaining (*A*_red_/*A*_nat_) at the indicated wavelength. Wavelengths indicated apply to all columns in each row, and sample headings in each column apply to all rows.

The above results regarding the impact of borohydride reduction on DOM absorbance are mainly focused on spectra. To explore how NaBH_4_ impacts the size distribution of absorbing material, Figure 6 plots absorbance chromatograms for native and reduced samples at multiple λ_*ex*_. A comparison of *A*_red_/*A*_nat_ as a function of elution volume is also shown for comparison, which reveals that higher molecular weight material (lower elution volume) has a greater fractional absorbance removal than lower molecular weight material. This finding is consistent with the lower *A*_red_/*A*_nat_ across the UV–visible at lower elution volumes (Figure 4). Although subtle, comparison of normalized chromatograms (Figure S3) indicates that reduced samples are slightly shifted to larger elution volumes.

To quantitatively describe changes in absorbance between native and reduced chromatograms, Figure S4 shows TCC and SSC values for native-reduced chromatograms compared to TCC and SSC for the column-no column control group. In the standard method for NaBH_4_ reduction, G-10 resin is used to remove borate.^12^ However, Sephadex is also a size exclusion resin (G-10 has an exclusion limit of 700 Da), and prior studies have not rigorously tested whether borate cleanup changes the molecular weight distribution of DOM. Only SRNOM and SRHA were processed with and without the G-10 column (see Text S3), but we consider the values for these samples to be a good threshold for evaluating TCC and SSC of other DOM samples. Based on TCC values, which are derived solely from peak position, one would conclude that there are minimal differences in chromatograms between the native and control groups. However, larger differences are seen when shape is considered. For example, SSC values for absorbance chromatograms for the column-no column group are >0.99 but tend to be lower for the native-control group, especially for SRHA, SRNOM, and ESHA (SSC < 0.98). This result indicates that NaBH_4_ reduction results in a shift in the size distribution of absorbing DOM at a range of λ_*ex*_, albeit exceedingly small.

### Impact of NaBH_4_ Reduction on DOM Fluorescence Spectra and Chromatograms

NaBH_4_ reduction results in increasing fluorescence intensity, despite the absorbance decrease, resulting in an increasing AQY. Increasing fluorescence emission and AQY has also been observed for NaBH_4_ reduction of bulk samples.^27,28^ Our results indicate that this increase in AQY occurs at a range of elution volumes (low to high), demonstrating that AQY increases for all molecular weights. This increase in AQY at a range of molecular weights is consistent with a prior study demonstrating that SRFA molecular weight fractions (<5K, bulk, and >5K) had similar enhancements in AQY at different λ_ex_ (between 2- and 3-fold).^60^

**Figure 7:**
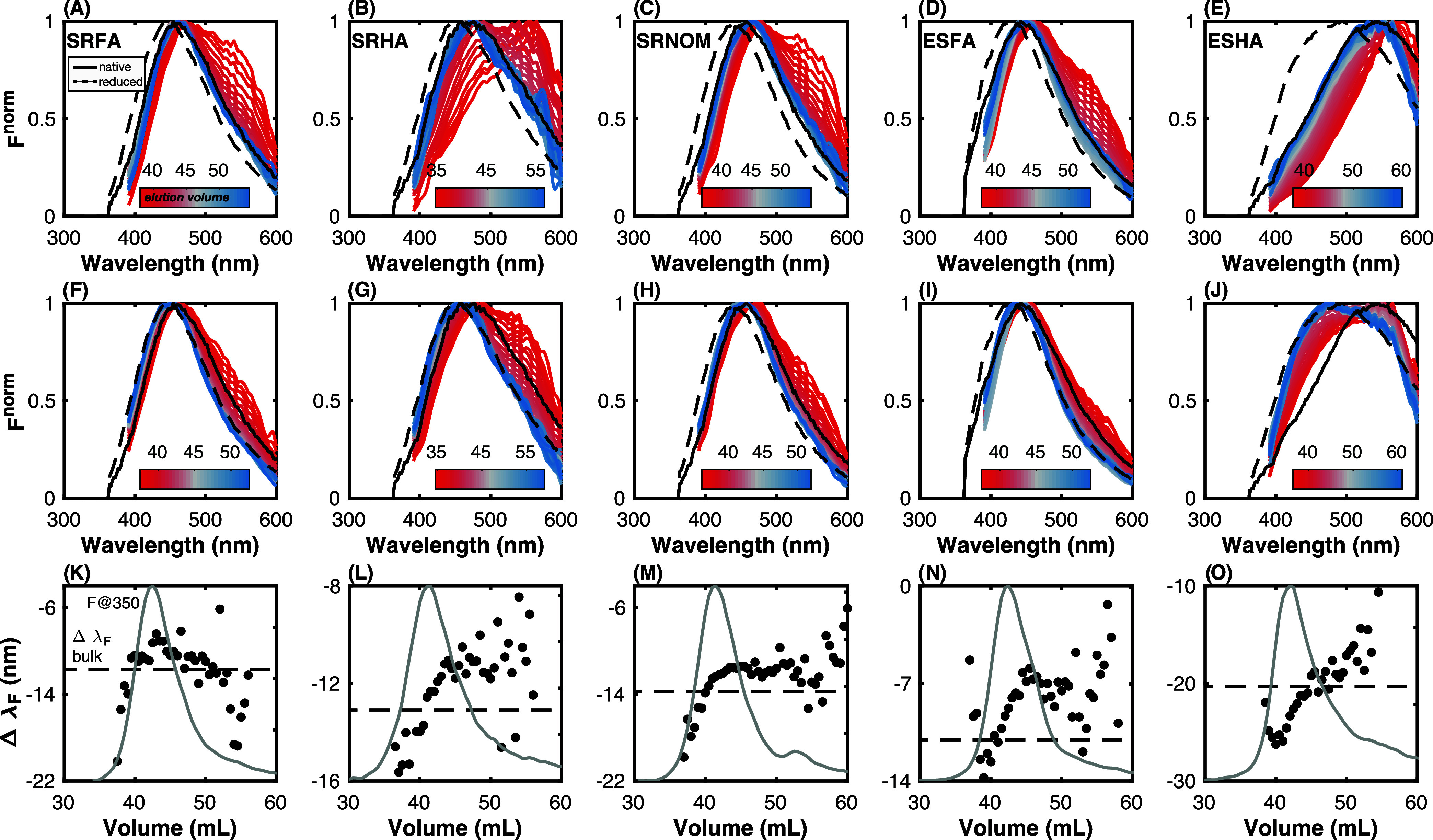
Fluorescence emission properties of DOM isolates as a function of molecular weight and reduction with NaBH_4_. Intensity-normalized emission spectra (F^norm^) of (A–E) native and (F–J) reduced samples, with the heatmap indicating elution volume. The solid and dashed lines in (A–J) represent the emission spectra of the native and reduced bulk samples, respectively. (K–O) Intensity-weighted difference in fluorescence emission wavelength (Δλ*_F_*) as a function of elution volume. The dashed lines represent Δλ_F_ for the bulk sample. The chromatogram of integrated emission at 350 nm excitation is depicted by the gray line (arbitrary *y*-axis). The sample labels in (A–E) apply to (F–O).

To further explore the impact of molecular weight and NaBH_4_ reduction on fluorescence spectral shape, Figure 7 shows the intensity-normalized emission spectra collected as a function of elution volume with the SEC fluorescence detector for native (Figure 7A–E) and reduced samples (Figure 7F–J). Spectra for the bulk native and reduced samples are shown for comparison. There are several interesting findings in Figure 7. First, emission spectra of the bulk samples more closely resemble spectra of material eluting at longer elution volumes, which can be explained by the higher AQY of lower molecular weight DOM. Second, there are large differences in peak emission maxima between the minimum and maximum elution volumes for each sample (e.g., > 50 nm for SRHA), with higher molecular weight exhibiting more red edge emission. Notably, the relative intensity of red edge emission is diminished in the reduced samples, suggesting that the emitting species responsible for this feature contain borohydride-reducible moieties. Third, for lower molecular weight material (e.g., at elution volumes greater than ∼45 mL), there is little change in emission maxima with elution volume. In contrast, spectra collected at lower elution volumes (high molecular weight) show a gradual blue-shift as the intensity of the ∼550 nm shoulder peak is diminished. Several prior reports using SEC, asymmetric field flow fractionation, and ultrafiltration have also demonstrated that higher molecular weight DOM tends to have red-shifted emission.^51,61^ Finally, all samples besides SRFA exhibited a clear trend of increasing Δλ*_F_* with increasing elution volume, with good agreement between Δλ_F_ for bulk and SEC samples. This shows that higher molecular weight material’s emission spectra is more blue-shifted after NaBH_4_ reduction compared to low molecular weight material.

In terms of chromatographic shape, the difference in SSC values between the column-no column and native-reduced groups is even more pronounced for fluorescence chromatograms (Figures S5 and S6) than absorbance chromatograms, especially for SRHA and ESHA. SSC values for the column-no column control group are near 0.99, while values for the native-reduced group are less than 0.98 and, in some cases, much lower (e.g., ESHA has SSC < 0.95). In contrast to the largely unidirectional shift in TOC and absorbance chromatograms to lower elution volumes, fluorescence chromatograms of reduced samples are slightly wider than native samples thus exhibiting increased signals (when both chromatograms are normalized to peak intensity) at both lower and higher elution volume. Collectively, these results indicate that the molecular weight of absorbing and emitting material at specific excitation and emission wavelengths is not appreciably changed by NaBH_4_ reduction.

## Size Dependence of Optical Surrogates for Native and Reduced Samples

SEC coupled to absorbance and fluorescence detectors provides an opportunity to test the underlying relationship between optical surrogates and DOM molecular weight. As highlighted recently,^62^ many of these optical surrogates originated in specific contexts that may not be broadly applicable to others. One of the most well-tested contexts is size separation of a single sample, mostly by ultrafiltration,^63,64^ with some studies also calculating optical surrogates from absorbance and fluorescence spectra collected during SEC.^51,61,65^

**Figure 8:**
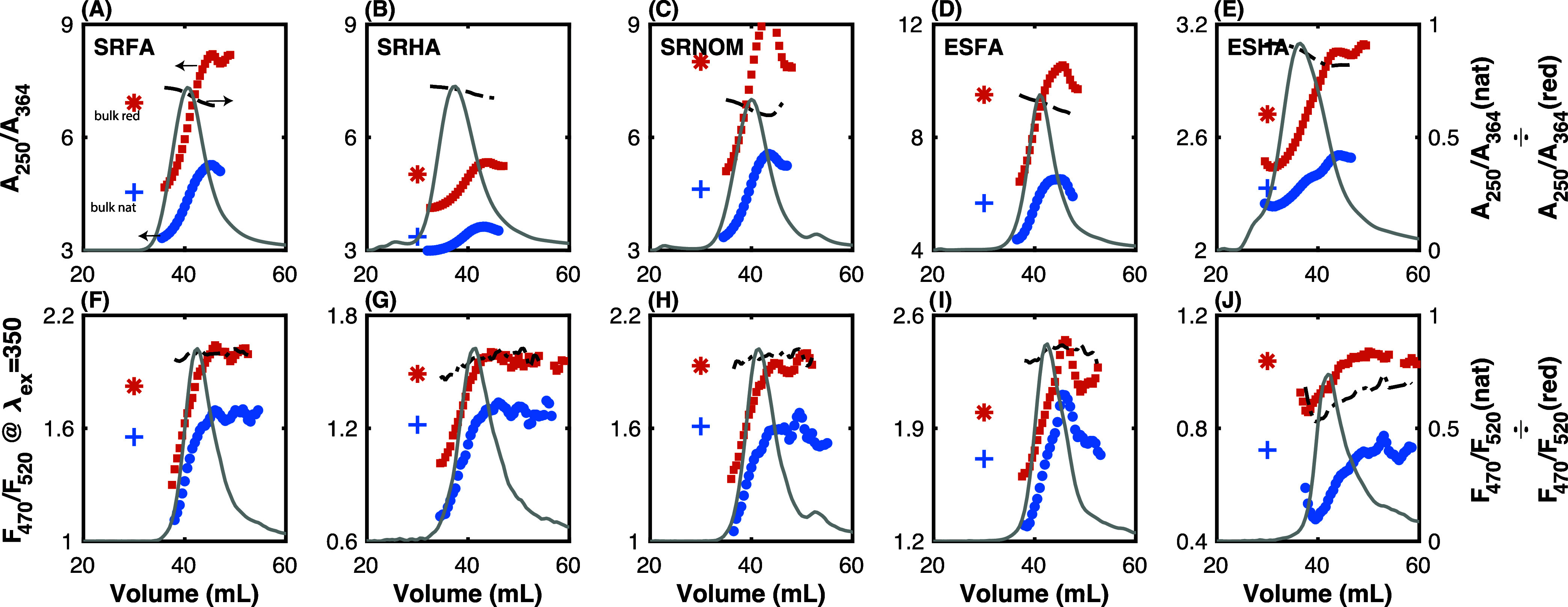
Dependence of optical surrogates on molecular weight for native and reduced DOM isolates. (A–E) *A*_250_/*A*_364_ (similar to E2:E3) as a function of elution volume. The chromatogram of absorbance at 250 nm is depicted by the gray line (arbitrary *y*-axis). (F–J) *F*_470_/*F*_520_ at 350 nm excitation (similar to fluorescence index, FI). Blue circles and orange squares represent native and reduced, samples, respectively. The normalized chromatogram of integrated emission at 350 nm excitation is depicted by the gray line (arbitrary *y*-axis). Optical surrogates for bulk native and reduced samples are indicated by + and *, respectively. The dotted black lines (− - – ) correspond to the ratio of surrogates for the native and reduced samples (right *y*-axis). The sample labels in (A–E) apply to (F–J).

Here, we focus on one absorbance-based surrogate (*A*_250_/*A*_364_, similar to E2:E3) and one fluorescence-based surrogate (*F*_470_/*F*_520_ collected at 350 nm excitation, similar to FI). *A*_250_/*A*_364_ and *F*_470_/*F*_520_ for native and reduced samples are shown in Figure 8, with values from bulk sample shown for comparison. Both *A*_250_/*A*_365_ and *F*_470_/*F*_520_ increase with decreasing molecular weight (larger elution volume), consistent with several continued inquiries employing different size separation techniques (SEC,^51,66^ asymmetric field flow fractionation,^61^ and ultrafiltration^63,64^), providing support that these optical surrogates are good indicators of molecular weight for a single, size-fractionated sample. Perhaps more interesting is that there appears to be a limit beyond which further decreases in molecular weight (i.e., increasing elution volume) do not result in increased *A*_250_/*A*_365_ and *F*_470_/*F*_520_. These results indicate that there is a subset of DOM molecules that violate traditional optical surrogate-molecular weight interpretations. A similar observation (*S*_275–295_ and *S*_350–400_ did not vary at <800 Da) was reported for Canadian river water samples by Guéguen and Cuss.^61^

Given that prior studies have demonstrated a strong relationship between FI and the emission maximum,^58,67−69^ we calculated the intensity-weighted emission wavelength at 350 nm excitation  and plotted these values against *F*_470_/*F*_520_ (Figure S7). There is a tight relationship in the *F*_470_/*F*_520_-emission wavelength curve that transcends DOM isolate origin (soil vs aquatic) and NaBH_4_ reduction status (native vs. reduced), resulting in a continuous exponential decrease in *F*_470_/*F*_520_ with increasing peak emission wavelength.

The continuous exponential relationship between *F*_470_/*F*_520_ and intensity-averaged emission maximum motivated us to evaluate whether there was an underlying mathematical explanation. To address this question, we modeled DOM fluorescence spectra using a Gaussian function and then took the ratio of fluorescence intensity at two wavelengths. The result of this derivation shown in eq S2.2 confirms the exponential relationship can be predicted mathematically (see also Text S4).

While the relationship between *F*_470_/*F*_520_ and intensity-averaged emission maximum is shown in part to be a mathematical result, the relationship between AQY and emission maximum has a fundamental photophysical basis. The fluorescence quantum yield (Φ_*f*_) can be written as the rate of radiative decay divided by the total rate of singlet excited state decay, Φ*_f_* = *k_f_*/*(k_f_* + *k*_*ic*_ + *k*_*isc*_) where *k_f_*, *k_ic_*, and *k_isc_* are rate constants for fluorescence, internal conversion, and intersystem crossing, respectively. Typically >90% of singlet excited state DOM undergoes internal conversion.^39^ The energy gap law states that the rate constant for internal conversion is an exponential function of the energy difference between the first excited electronic state (S_1_) and ground state (S_0_), .^70^ Being a mixture of absorbers, we use the intensity-weighted emission maximum as a surrogate for Δ*E*_*S*_1_→*S*_0__;^16^ the latter is normally determined by the mirror image rule. Thus, the energy gap law predicts that the AQY will decrease exponentially as a function of emission maximum. Consistent with this prediction, an exponential relationship between AQY and average emission wavelength is observed for most DOM isolates, both native and reduced (Figures S7 and S8). Notable exceptions to this relationship exist. For example, native ESFA shows a region (460–470 nm) in which AQY is insensitive to emission maximum. ESHA also shows behavior different than the Suwannee River isolates. Reduced ESHA exhibits a large range of AQY values over a very small emission wavelength range (500–510), and native ESHA has a large emission wavelength region (510–525 nm) in which AQY is constant. This bifurcation in emission properties of ESHA from other DOM isolates has been noted before in prior studies focused on solvation of DOM isolates in nonpolar organic solvents,^41^ borohydride reduction,^28^ and fluorescence quenching with cationic nitroxide free radicals.^71^

## Discussion

Here, we discuss the primary results of this study in the context of models for the three-dimensional structure of DOM and its relation to DOM optical properties.

Reduction of five DOM isolates by NaBH_4_ resulted in statistically significant but exceedingly small decreases in molecular weight observed by TOC and absorbance detectors. NaBH_4_ reduction converts carbonyls to alcohols, increasing molecular weight by 1 amu. Although minimal, a distinct shift to higher elution volume in TOC chromatograms is observed between 40–50 mL of all DOM isolates, which indicates a selective molecular weight decrease by NaBH_4_ reduction. The TOC shift is small compared to those typically observed during other chemical (e.g., ozone)^48,72,73^ and physical (e.g., ultrafiltration)^64^ treatments. Therefore, it is reasonable to conclude that the carbonyl-to-alcohol conversion and any disrupted electron donor–acceptor complexes (Scheme 1C) play a minimal role in DOM secondary structure. Although we cannot completely rule out that intermolecular electron donor–acceptor complexes may be influencing DOM three-dimensional structure, their overall contribution is likely small compared to other intermolecular forces. Consistent with this, a study by Wünsch et al.^51^ demonstrated a high degree of similarity in fluorescence spectral shape of bulk and SEC-separated DOM, suggesting a minimal role for intermolecular interactions in the formation of DOM fluorescence. On the other hand, the contribution of intramolecular charge-transfer interactions should also be taken into consideration. Disruption of intramolecular complexes could be expected after borohydride reduction, but the impacts on SEC results would be more difficult to predict. Loss of intramolecular interactions would not decrease molecular weight, but could enable more degrees of freedom that could be manifested in an increased apparent size measured by SEC.

It should be noted that one prior study employing dynamic light scattering (DLS) demonstrated an increase in the average diameter of Aldrich Humic Acid reduced with NaBH_4_ relative to the native sample.^74^ Given prior problems noted with commercial humic acids as a model for humic substances (e.g., interbatch consistency, unreported ash content, and lack of information about source and isolation),^75^ we believe the results shown here and those for Aldrich Humic Acid^74^ are not necessarily in conflict.

Our results suggest that borohydride-reducible moieties (i.e., aromatic ketones or aldehydes, and quinones) are present throughout DOM’s molecular weight distribution and not isolated in one size class. This conclusion is also supported by Tinnacher and Honeymann^26^ who demonstrated a near uniform distribution in radioactivity after labeling SRFA with ^3^H via tritiated NaBH_4_. That being said, changes in optical properties of larger sized molecules were more pronounced, such as lower A_red_/A_nat_ and more negative shifts in Δλ_*F*_. The origin of these more significant changes is currently unclear. One model contends that borohydride-reducible charge-transfer contacts are partially isolated in a hydrophobic core,^40,42^ which seems inconsistent with our results. However, it is still possible that solvent-inaccessible borohydride-reducible moieties exist if there are enough solvent-accessible reduction sites. If true, this would suggest that higher molecular weight DOM contains a greater concentration of carbonyl-containing chromophores. Future work could address this question by solid-state ^13^C NMR analysis of high molecular weight DOM prepared by ultrafiltration.

Finally, our results contribute to the existing literature supporting optical measurements as surrogates for DOM molecular weight.^62^ Importantly, the correlations observed between optical surrogates and molecular weight (via elution volume) are for five DOM isolates, native and reduced, developed over a fine size gradient achieved by SEC. Indeed, comparing optical surrogates for native and reduced values demonstrates that other variables besides molecular weight are impacting these surrogates. Although reduced DOM isolates do exhibit a very slight decrease in molecular weight (Figures 2, S3 and S4), the changes in optical surrogates are much more pronounced. In other words, it is likely that modification of the underlying chromophores is responsible for the increase in *A*_250_/*A*_364_ and *F*_470_/*F*_520_, not shifts in molecular weight. Consistent with several prior investigations^51,61,76−82^ is the observation that absorbance and fluorescence spectral shape converge at lower molecular weights, which is reflected in the constancy of both *A*_250_/*A*_364_ and *F*_470_/*F*_520_ at >45 mL of the SEC chromatogram. This result implies that low molecular weight DOM may be insensitive to further spectral shifts, decreasing optical surrogate’s utility for monitoring molecular weight below a certain limit.

## Conclusions

By studying the impact of NaBH_4_ reduction using size exclusion chromatography coupled to TOC, absorbance, and fluorescence detectors, we demonstrated that

1. Reduction with NaBH_4_ results in quantifiable yet exceedingly small decreases in the apparent size of DOM, indicating that intermolecular interactions involving borohydride-accessible carbonyl-containing moieties play a minimal role in modulating the three-dimensional structure of DOM.
2. Borohydride-reducible groups are present in all size fractions of DOM. However, high molecular weight DOM may have a higher proportion of these groups based on the more pronounced changes in absorbance spectra (A_red_/A_nat_) and fluorescence emission blue shift at lower elution volumes.
3. The energy gap law is suggested as the photophysical mechanism responsible for the inverse dependence of AQY on emission maximum for DOM of varying size.
4. Optical surrogates are highly sensitive to NaBH_4_ reduction despite minimal shifts in DOM molecular weight.

These results provide several potential avenues for future work. First, it would be of interest to perform NaBH_4_ reduction experiments on size-fractionated DOM (e.g., by ultrafiltration) and then subject samples to SEC analysis. Second, the relative wavelength independence of A_red_/A_nat_ for high molecular weight material calls into question the widely stated concept that preferential removal of visible absorption by borohydride reduction is evidence of charge-transfer interactions. Follow up studies could test this hypothesis by measuring A_red_/A_nat_ for model organic chromophores known to absorb/emit locally and as donor–acceptor complexes. Third and finally, additional studies are needed to support the mechanism responsible for the small decrease in molecular weight following borohydride reduction. Our hypothesis is that conversion of carbonyl moieties to alcohols results in loss of charge-transfer contacts that decreases DOM molecular weight. This could be tested experimentally using additional size characterization techniques (e.g., asymmetric field flow fractionation, electron microscopy) or computational approaches such as molecular dynamics.

## Data Availability

Data for this article is available in the main manuscript tables and figures or in the Supporting Information. Other data will be made available to upon reasonable request to the authors.

## Supporting Information Available

Additional discussions, tables, and figures; see SI table of contents for specific details (PDF)
