# Supplementary material for: Characterization of Natural Organic Matter and Humic Substance Isolates by Size Exclusion Chromatography following Reduction with Sodium Borohydride
Source: ACS Environ Au. 2024 Dec 20;5(2):197–210. doi: 10.1021/acsenvironau.4c00075 (PMC11926756; doi:10.1021/acsenvironau.4c00075)
Supplement: Supplementary file 1 — vg4c00075_si_001.pdf [file vg4c00075_si_001.pdf]

Supporting Information for

Characterization of Natural Organic Matter and Humic Substance Isolates by Size Exclusion Chromatography following Reduction with Sodium Borohydride

Hang Li[a], Blair Hanson[b,c], and Garrett McKay[a]*

[a]Zachry Department of Civil & Environmental Engineering, Texas A&M University, College Station, TX, 77843, USA

[b]Department of Civil, Environmental, and Architectural Engineering and [c]Environmental Engineering Program, Univeresity of Colorado Boulder, Boulder, CO, 80303, USA

*Corresponding author
Email: gmckay@tamu.edu
Phone: (979) 458-6540

Number of Pages: 11

Number of Text Sections: 5

Number of Figures: 9

Text S1 – Quality control of fluorescence data

In our initial attempt at developing quality control criteria for fluorescence spectra collected during SEC, we used the approach developed by Korak and McKay (2024). Briefly, this approach creates smoothed emission spectra from the data using loess and then calculates the root mean squared error between smoothed and measured data. However, this approach resulted in quality control criteria that excluded most measured spectra and was thus deemed unacceptable. This result is a symptom of the greater noise of emission spectra measured by the Agilent FLD relative to stand-alone benchtop spectrofluorometers.

Once we decided on using a traditional signal-to-noise approach (see main manuscript), which required back-calculating fluorescence intensities prior to inner filter effect corrections and instrument-specific corrections, we considered using a criterion in which signals in a chromatogram less than the QC threshold were selectively removed. However, this approach resulted in non-uniform data matrix dimensions between native and reduced samples. TCC and SSC require matrices of the same size, so this approach was deemed unacceptable.

Ultimately, we arrived at the approach described in the manuscript, where chromatograms with maxima greater than 20 times the average background noise (95th percentile) were retained.

Text S2 – Purpose of Sephadex G-10 columns as a TCC and SSC control

SRFA and SRHA were tested with and without Sephadex G-10 column removal. Because early experiments demonstrated no meaningful differences in SEC chromatograms between native NOM samples treated and not treated with Sephadex G-10, we chose not to subject additional samples to G-10 fractionation. This explains how the column-no column group is used as a TCC and SSC benchmark.

Text S3 – Rationale for not calibrating SEC elution volume to molecular weight

According to the methods of Her et. al.,[1] the SEC system was initially calibrated using polyethylene glycols (PEGs) and was used to verify that molecular weight estimations of this system agree with that of previous studies.[2] However, DOM is a complex mixture of compounds with a high degree of variability in molecular structure, where calibration standards are uniform, monodisperse compounds. Previous studies have demonstrated that structural differences exist between size-based fractions.[3,4] Additionally, fractionation by SEC is based on molecular size, rather than molecular weight and sample elution is dependent upon parameters including column material (i.e., stationary phase), and mobile phase composition (e.g., pH and ionic strength). Reported molecular ranges are highly variable, in some cases differing by thousands of Daltons.[5] As a result, calibration can only provide estimated apparent molecular weights, and estimations obtained from different SEC systems should not be directly compared. Therefore, results described in this study are given in terms of elution volume where our conclusions can be fully supported by relative trends in molecular weights. Our choice is in line with other previous SEC studies that also reported results in terms of elution volume.[6,7]

Text S4 – Relationship between F_{470}/F_{520} and intensity-weighted emission average

As we dived deeper into the relationship between optical surrogates, we found that the intensity-weighted average λ_{em} ($\lambda_{em,avg}$) has a near negative exponential relationship to both the ratio of emission intensity F_{470}/F_{520} and the apparent quantum yield (AQY) at λ_{ex}=350 nm. (Figure S7) While the relationship $\lambda_{em,avg}$ and AQY is dependent on the intrinsic optical property of DOM fractions, F_{470}/F_{520} is mathematically related to $\lambda_{em,avg}$.

Since the emission spectra of DOM fractions at a single excitation wavelength are similar in shape to Gaussian curves (Eqn. S1.1), they can be fit to such curves with three constants: a, height of the peak, b, peak position on x-axis, and c, the width of the curve, with b also equivalent to $\lambda_{em,avg}$. (Figure S9, Eqn. S1.2) F_{470}/F_{520} as a ratio of two $f(x)$ values can also be solved with the Gaussian function with known x values being 470 nm and 520 nm. (Eqn. S2.1, 2.2) Here F_{470}/F_{520} is an exponential function to negative $\lambda_{em,avg}$, and also c^2, square of curve width. Even though the emission spectra have various curve widths for DOM fractions, most c's happen to be around 60 when we fit them to Eqn. S1.2. Figure S9 (right) shows a collective relationship between F_{470}/F_{520} and $\lambda_{em,avg}$, which can be fit to Eqn. S2.2 with a c value of 45.2.

$$f(x) = ae^{\frac{-(x-b)^2}{2c^2}} \tag{S1.1}$$

$$I(\lambda_{em}) = ae^{\frac{-(\lambda em-\lambda em,avg)^2}{2c^2}} \tag{S1.2}$$

$$\frac{F(\lambda_1)}{F(\lambda_2)} = \frac{ae^{\frac{-(\lambda_1-\lambda_{em,avg})^2}{2c^2}}}{ae^{\frac{-(\lambda_2-\lambda_{em,avg})^2}{2c^2}}} = \exp\left(\frac{(\lambda_2-\lambda_1)(\lambda_2+\lambda_1-2\lambda_{em,avg})}{2c^2}\right) \tag{S2.1}$$

$$\frac{F(470nm)}{F(520nm)} = \exp\left(\frac{(520nm-470nm)(520nm+470nm-2\lambda_{em,avg})}{2c^2}\right) = \exp\left(\frac{(50nm)(990nm-2\lambda_{em,avg})}{2c^2}\right) \tag{S2.2}$$

Text S5 - Uniform maximum at A_{red}/A_{nat} and F_{red}/F_{nat}

Another interesting feature of both absorbance and fluorescence SEC is the local maximum of reduced to native ratio around 50 mL. (Figure S2, S5). The ratio describes the proportion of absorbance/emission intensity influenced by borohydride reduction, which is negatively related to the elution volume until 50 mL, then the opposite. Despite the differences of DOM isolates in their composition, they all present a peak reduction-resistance feature in their fractions around 50 mL.

There are several potential explanations for the phenomenon. First, borohydride-reducible groups distribute less around 50 mL. Even though ketones, aldehydes are

functional groups exist on both aliphatic and aromatic groups of different sizes, it is possible that saturated carbons gather more around 50 mL. A second possible explanation is the change of molecular weight of light absorbing and fluorescence emitting moieties which located >50 mL to 50 mL because of reduction. Since this is contrary to the theory that borohydride reduction caused breakage of DOM fractions, such explanation is less possible. A more likely explanation is, even though the influence of borohydride on absorbance/fluorescence is greater at larger fractions, there’s a particular group of moieties after 50 mL is so significantly reduced by borohydride, that the increasing trend of the ratio cuts off at 50 mL, which makes 50 mL a peak.

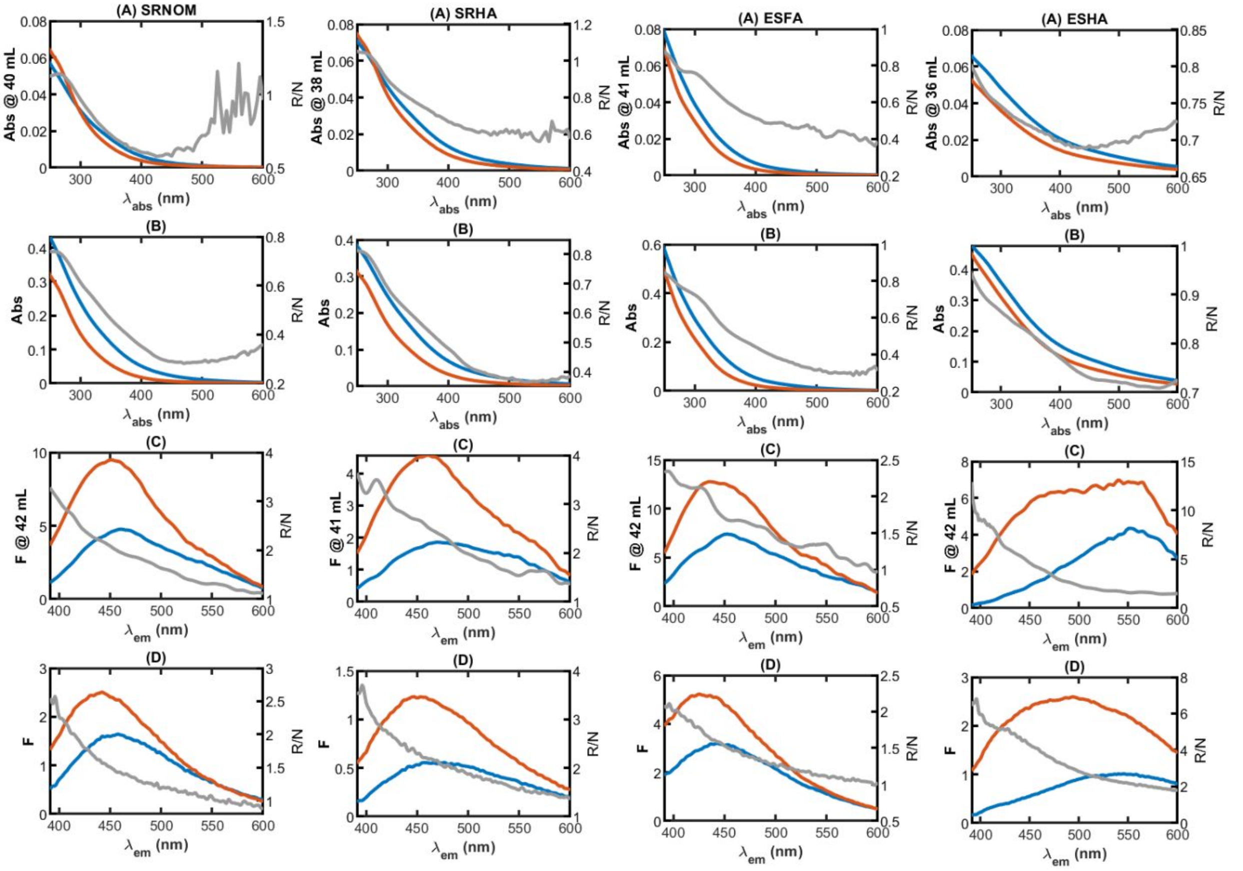

Figure S1. Absorbance (A and B) and fluorescence spectra (C and D) of DOM isolates at the peak elution volume (A and C) and bulk samples (B and D). The right y-axis (grey lines) shows the ratio of reduced to native spectra. Spectra in D) are normalized to water Raman scattering, whereas units in C) are arbitrary. Emission spectra in C) and D) are collected at the 350 nm excitation.

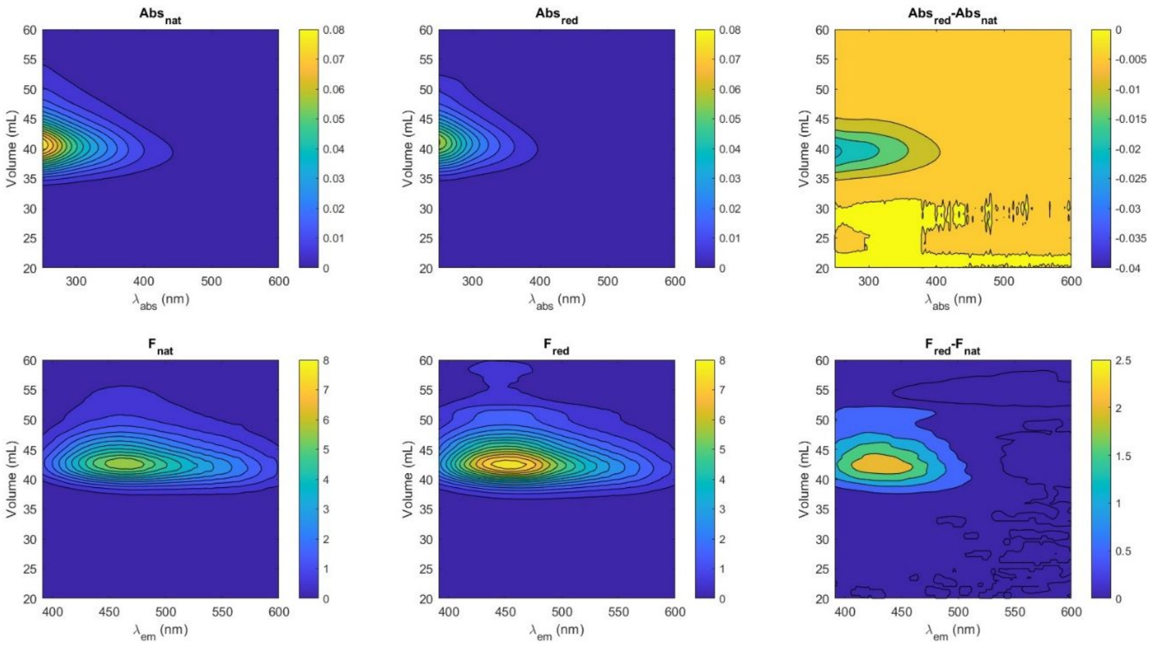

Figure S2. Three-dimensional contour plot of absorbance and fluorescence (350nm excitation) chromatography of native SRFA (left), reduced SRFA (middle) and their difference (right).

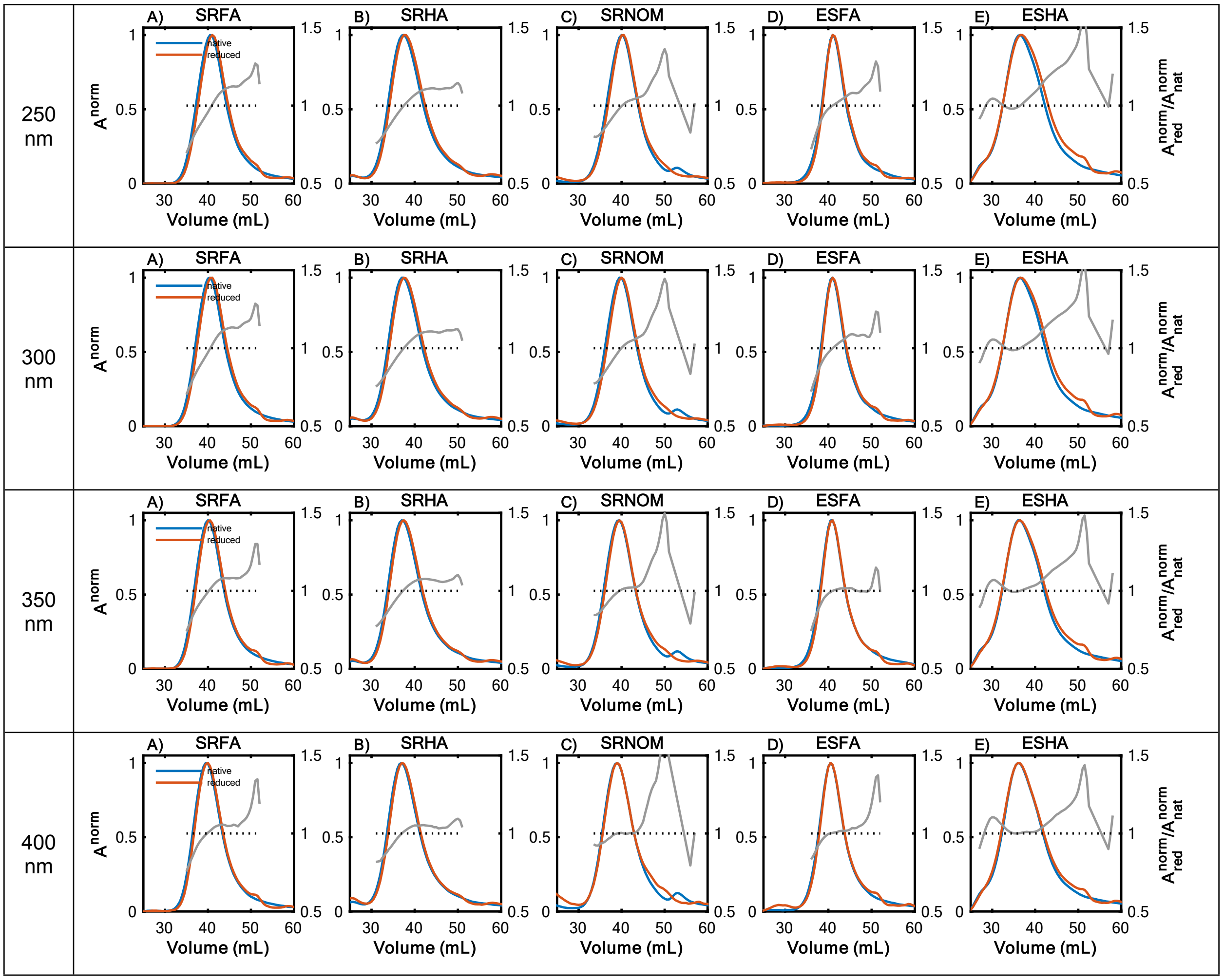

Figure S3. Absorbance chromatograms for all DOM isolates at varying wavelengths. Left y-axes correspond to peak-normalized absorbance chromatograms for either native or reduced samples (A^{norm}). Right y-axes correspond to the ratio of normalized reduced to native chromatograms.

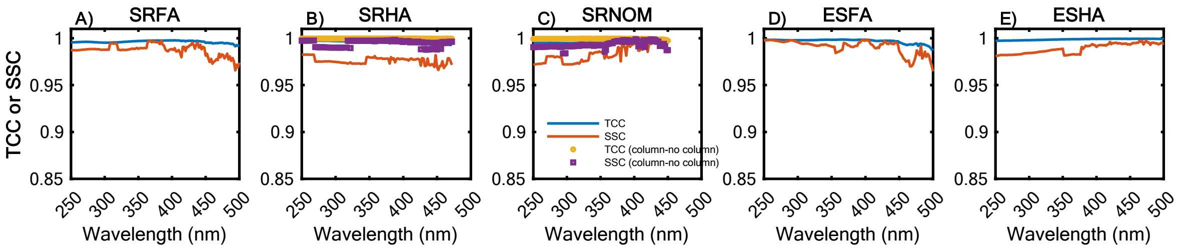

Figure S4. Tucker Congruence Coefficient (TCC) and shape sensitive congruence (SSC) comparing native and reduced absorbance chromatograms as a function of absorbance wavelength for DOM isolates. Markers indicate the control group (column-no column) whereas solid lines indicate the treatment group (native-reduced).

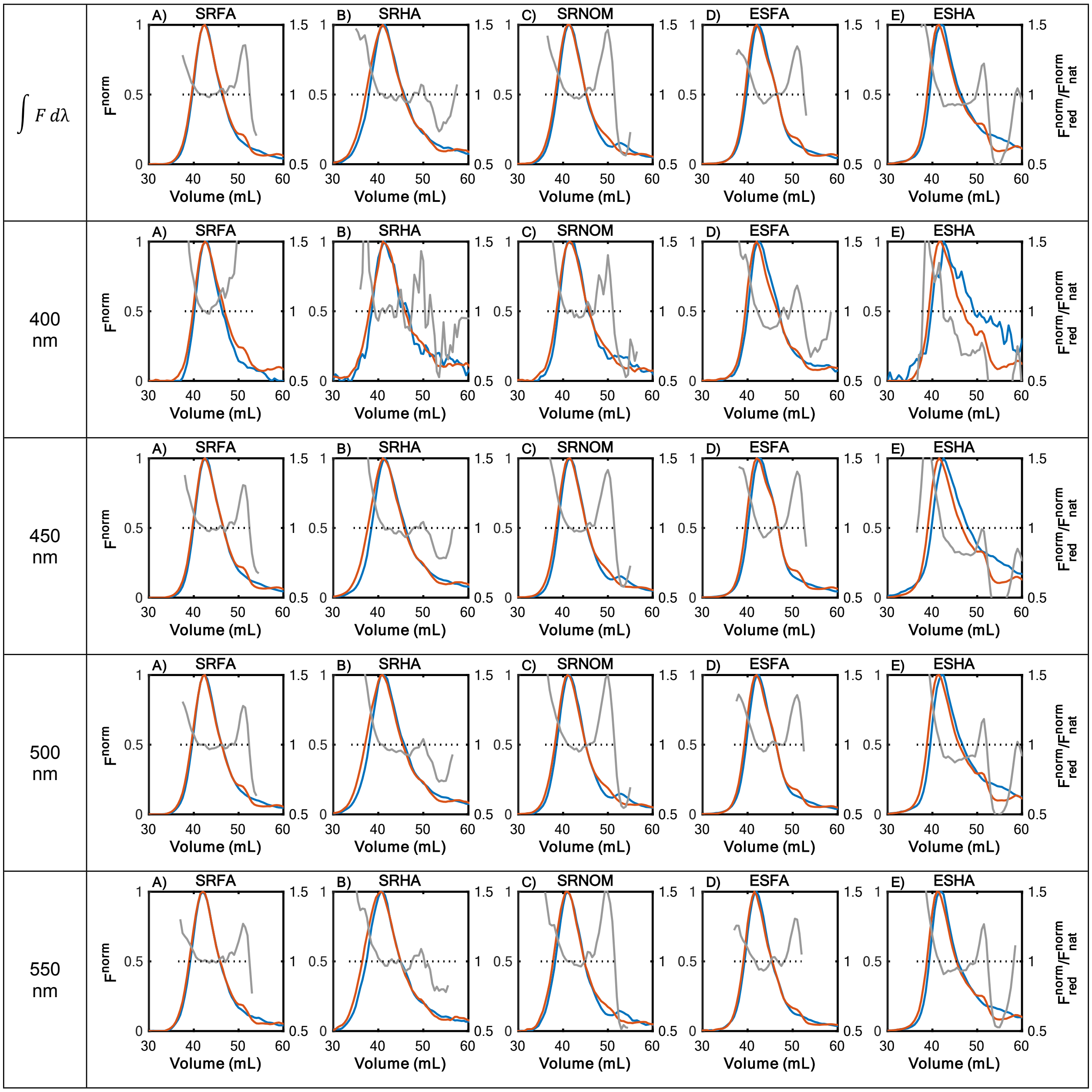

Figure S5. Fluorescence chromatograms for all DOM isolates at varying emission wavelengths or integrated emission (top row) measured at 350 nm excitation. Left y-axes correspond to peak-normalized fluorescence chromatograms for either native or reduced samples (F^{norm}). Right y-axes correspond to the ratio of normalized reduced to native chromatograms.

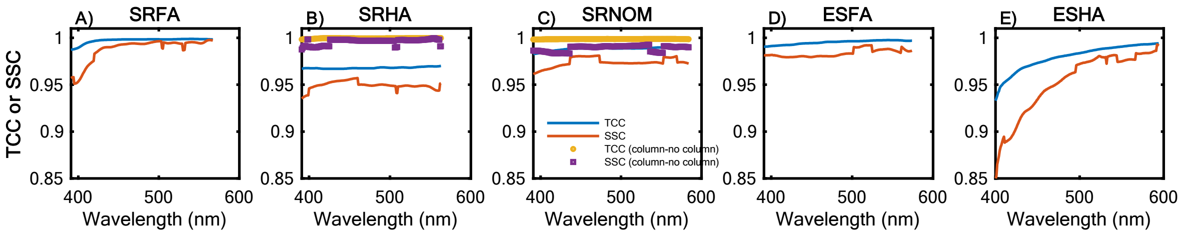

Figure S6. Tucker Congruence Coefficient (TCC) and shape sensitive congruence (SSC) comparing native and reduced fluorescence chromatograms as a function of fluorescence emission wavelength for DOM isolates. Markers indicate the control group (column-no column) whereas solid lines indicate the treatment group (native-reduced).

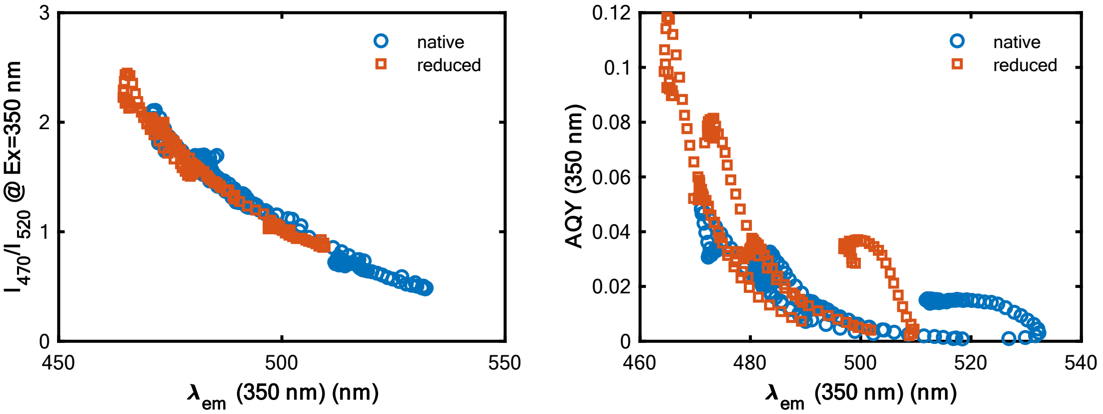

Figure S7. Relationship between the intensity-weighted emission wavelength and (left) the ratio of emission intensities at 470 to 520 nm (collected at 350 nm excitation) and (right) the apparent quantum yield (AQY).

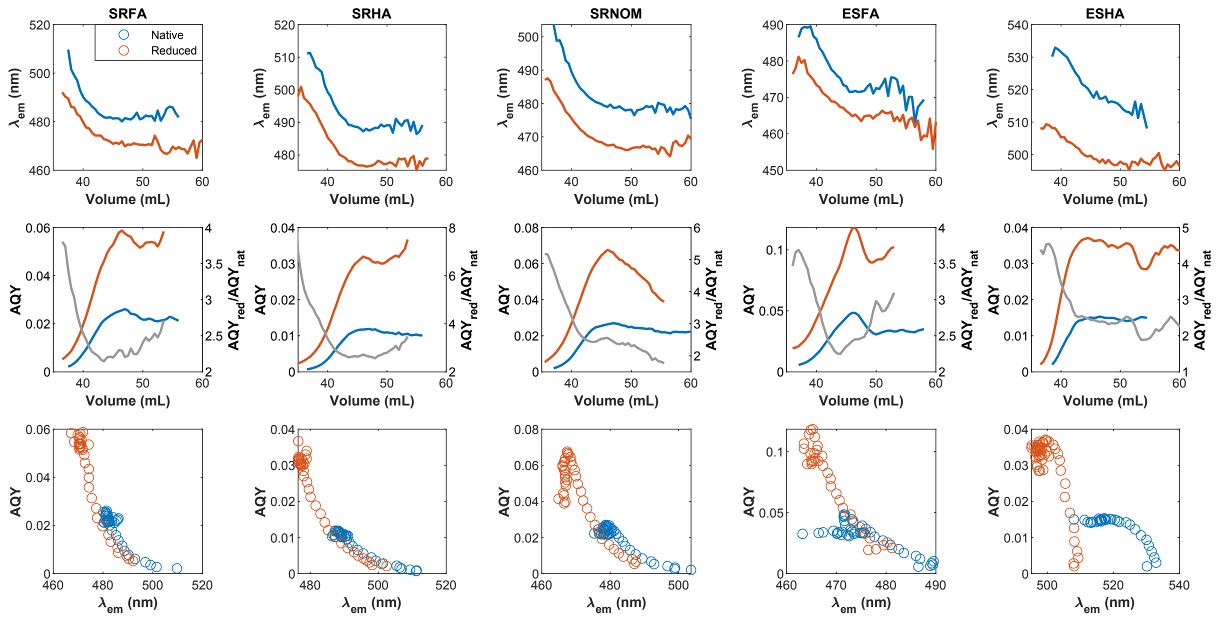

Figure S8. Row 1: the intensity-weighted emission wavelength at 350 nm excitation at each elution volume; Row 2: apparent quantum yield (AQY) at each elution volume; Row 3: relationship between the intensity-weighted emission wavelength at 350 nm excitation and AQY.

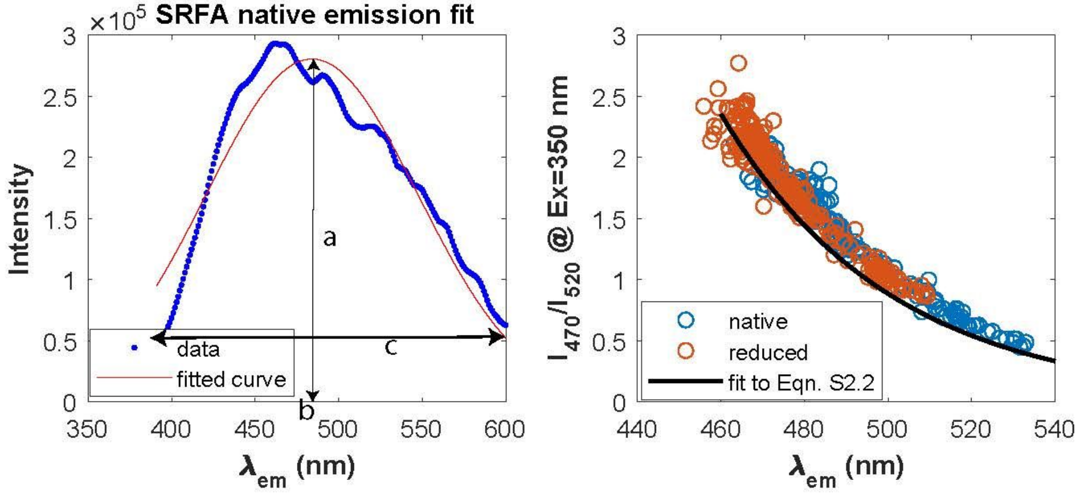

Figure S9. Left: SRFA native spectra at λex=350 nm, 40 mL elution volume, fit to gaussian curve Eqn. S1.1; Right: relationship between the intensity-weighted emission wavelength at 350 nm excitation and the ratio of emission intensities at 470 to 520 nm of all samples, and Eqn. S2.2 with c=45.2.

References

1. Her, N.; Amy, G.; Foss, D.; Cho, J., Variations of Molecular Weight Estimation by HP-Size Exclusion Chromatography with UVA versus Online DOC Detection. *Environmental Science & Technology* **2002,** *36* (15), 3393-3399.
2. Dong, M. M.; Mezyk, S. P.; Rosario-Ortiz, F. L., Reactivity of effluent organic matter (EfOM) with hydroxyl radical as a function of molecular weight. *Environmental Science & Technology* **2010,** *44* (15), 5714-5720.
3. Hawkes, J. A.; Sjöberg, P. J. R.; Bergquist, J.; Tranvik, L. J., Complexity of dissolved organic matter in the molecular size dimension: insights from coupled size exclusion chromatography electrospray ionisation mass spectrometry. *Faraday Discussions* **2019,** *218* (0), 52-71.
4. Huber, S. A.; Balz, A.; Abert, M.; Pronk, W., Characterisation of aquatic humic and non-humic matter with size-exclusion chromatography - organic carbon detection - organic nitrogen detection (LC-OCD-OND). *Water research* **2011,** *45* (2), 879-885.
5. Sandron, S.; Rojas, A.; Wilson, R.; Davies, N. W.; Haddad, P. R.; Shellie, R. A.; Nesterenko, P. N.; Kelleher, B. P.; Paull, B., Chromatographic methods for the isolation, separation and characterisation of dissolved organic matter. *Environ Sci-Proc Imp* **2015,** *17* (9), 1531-1567.
6. Buckley, S.; Leresche, F.; Hanson, B.; Rosario-Ortiz, F. L., Decoupling Optical Response and Photochemical Formation of Singlet Oxygen in Size Isolated Fractions of Ozonated Dissolved Organic Matter. *Environmental Science & Technology* **2023,** *57* (14), 5603-5610.
7. Hanson, B.; Wünsch, U.; Buckley, S.; Fischer, S.; Leresche, F.; Murphy, K.; D'Andrilli, J.; Rosario-Ortiz, F. L., DOM Molecular Weight Fractionation and Fluorescence Quantum Yield Assessment Using a Coupled In-Line SEC Optical Property System. *Acs Es&T Water* **2022,** *2* (12), 2491-2501.
